# Fluconazole-induced liver injury in patients with pulmonary cryptococcosis: a comprehensive study integrating clinical cohort analysis, network toxicology, molecular docking, and transcriptomics

**DOI:** 10.1128/aac.01976-25

**Published:** 2026-04-20

**Authors:** Yiding Xu, Lei Chen, Jie Liu, Qin Wang, Xiaodan Zhu, Minghui Wang, Jianghong Li, Lin Shi, Hongni Jiang

**Affiliations:** 1 Department of Pulmonary Medicine, Zhongshan Hospital, Fudan University 12478, Shanghai, China; 2 Department of respiration, Xi’an international medical center hospital, Xi’an, China; 3 Department of Pulmonary Medicine, Xiamen Branch, Zhongshan Hospital, Fudan University 580032 , Xiamen, China; University of Houston, Houston, Texas, USA

**Keywords:** fluconazole, hepatotoxicity, pulmonary cryptococcosis, cohort study, network toxicology, molecular docking, transcriptomics

## Abstract

Fluconazole is key for pulmonary cryptococcosis (PC), but its hepatotoxicity (HT) risk is unclear. This study characterized fluconazole-induced liver injury in HIV-negative PC patients and explored its mechanism. We integrated clinical cohort analysis (*n* = 123), network toxicology, molecular docking, and transcriptomics. HT incidence was 23.6%, predominantly cholestatic (55.2%). Immunocompromised status independently increased HT risk (aRR = 2.67, *P* = 0.004). Baseline ALT >16.5 U/L was the best predictor (AUC = 0.69) among liver enzymes, which showed modest discriminatory value. A model combining liver enzymes, immune status, and comedications achieved superior prediction (AUC = 0.76). Mechanistically, AKT1, ERBB2, and KDR were identified as potential core targets. Transcriptomics confirmed their significant downregulation in general cholestasis models, and molecular docking demonstrated favorable affinity binding with fluconazole (strongest for KDR: −6.5 kcal/mol). In conclusion, baseline liver enzymes and immune status are critical risk factors for fluconazole-induced HT, and fluconazole-induced cholestatic injury may involve the dysregulation of signaling pathways centered on AKT1, ERBB2, and KDR, suggesting potential targets for monitoring.

## INTRODUCTION

Pulmonary cryptococcosis (PC), an invasive fungal disease caused predominantly by *Cryptococcus neoformans* or *Cryptococcus gattii*, continues to impose substantial global morbidity and mortality burdens across both immunocompetent and immunocompromised populations (1, 2). The 2024 IDSA/ECMM guidelines recommend fluconazole monotherapy (400 mg/day for 6–12 months) as first-line treatment for mild-to-moderate isolated PC (3). However, emerging safety concerns persist regarding fluconazole-associated hepatotoxicity (HT), a potentially life-threatening adverse effect that requires systematic risk stratification.

Drug-induced liver injury (DILI) represents the leading cause of acute hepatitis and hepatic failure, with an annual incidence of 14–19 clinically significant cases per 100,000 population (4, 5). Approximately 10% of DILI cases progress to severe outcomes including acute liver failure or death (6). Diagnostic standardization remains contentious, with conflicting laboratory thresholds proposed by four major frameworks: Common Terminology Criteria for Adverse Events (CTCAE), the Council for International Organizations of Medical Sciences (CIOMS), Drug-Induced Liver Injury Network (DILIN), and International Drug-Induced Liver Injury Expert Working Group (DEWG) (7–9). Comparative analyses demonstrate that CIOMS criteria exhibit superior sensitivity for case identification, whereas DEWG and DILIN criteria provide enhanced specificity and diagnostic accuracy (10).

Azole-associated HT presents unique challenges due to prolonged treatment courses. A 2022 real-world study reported HT incidence rates of 19.4% for fluconazole, 14.5% for itraconazole, and 32.5% for voriconazole (11). Moreover, fluconazole demonstrates a severe HT rate of 6.1 events per 10,000 patient-years (12), which may delay therapy or provoke fatal hepatic failure. Chronic liver disease has been identified as a critical risk amplifier for azole-related liver injury (13), while the immunocompromised status prevalent in 60% of PC patients (2) may further modulate hepatotoxic susceptibility.

Mechanisms underlying liver injury induced by several azole antifungals have been extensively investigated. Evidence from quantitative systems toxicology and plasma metabolomics supports a close association between voriconazole-induced liver injury and oxidative stress (14, 15). Liver injury associated with ketoconazole may involve the overexpression of sterol regulatory element-binding transcription factor 1 (16). Furthermore, ketoconazole and itraconazole induce liver injury potentially through activation of cytochrome P450 enzymes (17, 18). However, the mechanism underlying fluconazole-associated HT remains unclear.

Network toxicology represents an emerging approach that facilitates a comprehensive elucidation of interactions between toxic chemicals and biological network systems within organisms (19). Molecular docking, an advanced computational method, is pivotal for predicting the binding interactions between molecules and their biological targets (20). In recent years, an increasing body of research has integrated network toxicology, molecular docking, and transcriptomics to conduct multidimensional exploration of the toxicological mechanisms underlying chemical substances.

Therefore, we conducted a comprehensive study on fluconazole-associated HT. By integrating clinical cohort analysis, network toxicology, molecular docking, and transcriptomics, this study reveals the characteristics, risk factors, and molecular mechanisms underlying fluconazole-induced liver injury in patients with PC, aiming to better predict and treat this adverse effect.

## MATERIALS AND METHODS

### Study design and participants

**Fig 1:**
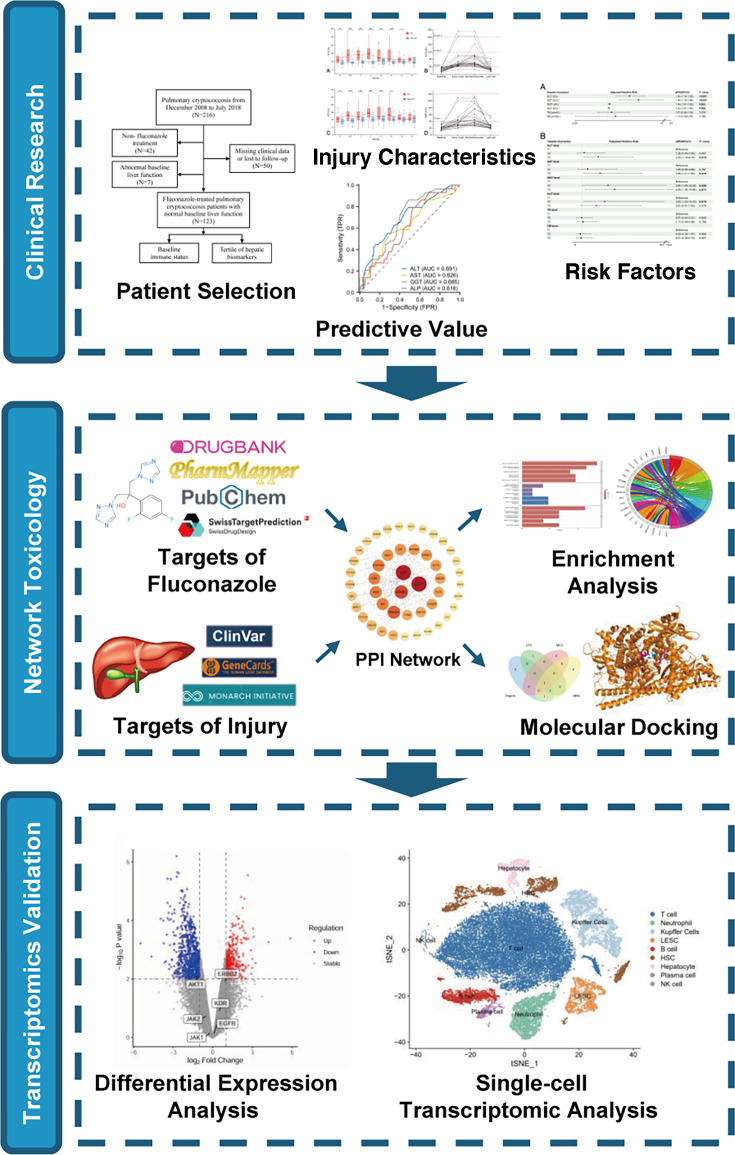
The flowchart of this study.

**Fig 2:**
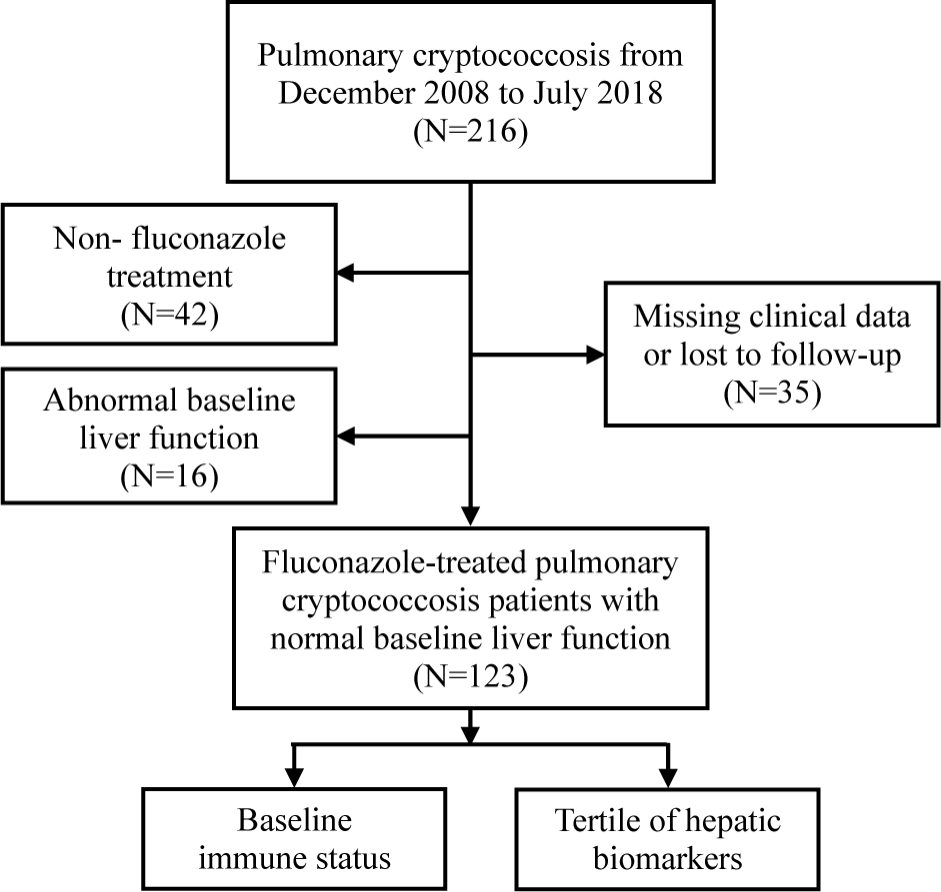
The selection and classification of participants.

The research process of this comprehensive study was shown in Fig. 1. The retrospective cohort study analyzed HIV-negative PC patients diagnosed and treated at Zhongshan Hospital, Fudan University, between December 2008 and July 2018. Patients with non-fluconazole treatment or without complete clinical data and follow-up information were excluded (Fig. 2).

### Data collection

Demographic characteristics, comorbidities, clinical manifestations, and medication histories were retrieved from electronic health records. Baseline hepatic function markers included alanine aminotransferase (ALT), aspartate aminotransferase (AST), gamma-glutamyl transpeptidase (GGT), alkaline phosphatase (ALP), total bilirubin (TB), and direct bilirubin (DB). Immunocompromised status was defined as meeting ≥1 of the following criteria within 6 months preceding fluconazole administration: active malignancy receiving chemotherapy, glucocorticoid/immunosuppressant use, autoimmune diseases, immunodeficiency disorders, or neutropenia (absolute neutrophil count <1.5 × 10/L). Data extraction was performed independently by two investigators using standardized case report forms. All discrepancies were resolved through consensus discussions to ensure data accuracy.

### Follow-up and definition of hepatic dysfunction

Longitudinal clinical data encompassing hepatic function profiles (including ALT and AST), antifungal medication regimens (specific agents and dosages), concomitant medications, and treatment outcomes of PC were systematically recorded during follow-up assessments. The association between liver function biomarkers, in both continuous and tertile forms, and fluconazole-induced HT was discussed separately to explore linear or non-linear relationships.

Fluconazole-associated HT was defined as a documented elevation of liver enzymes meeting any of the four criteria below. Four diagnostic frameworks were employed to assess DILI:

- CTCAE criteria: serum ALT or AST exceeding 40 U/L (>1 × upper limit of normal [ULN]);
- CIOMS criteria required fulfillment of at least one of the following: ALT > 2 × ULN, DB > 2 × ULN, or AST/ALP/TB exceeding 1 × ULN with at least one parameter > 2 × ULN;
- DILIN criteria mandated either two consecutive measurements of ALT/AST > 5 × ULN or ALP > 2 × ULN, TB > 1.4 × ULN with concurrent elevation of ALT/AST/ALP, or international normalized ratio (INR) > 1.5 alongside abnormal ALT/AST/ALP;
- DEWG criteria defined liver injury as ALT ≥ 5 × ULN, ALP ≥ 2 × ULN, or ALT ≥ 3 × ULN combined with TB ≥ 2 × ULN.

HT severity was graded by CTCAE as follows: Grade 1 (ALT/AST > 1–3 × ULN), Grade 2 (>3–5 × ULN), Grade 3 (>5–20 × ULN), and Grade 4 (>20 × ULN). HT patterns were classified using the serum biomarker ratio *R* = (ALT/ULN)/(ALP/ULN), with *R* ≤ 2 indicating cholestatic injury, *R* ≥ 5 indicating hepatocellular injury, and 2 < *R* < 5 indicating mixed injury.

### Screening of potential targets of fluconazole

We initiated our target identification by retrieving the two-dimensional structure and SMILES sequence of fluconazole from the PubChem database (https://pubchem.ncbi.nlm.nih.gov/). Subsequently, we employed three complementary target prediction platforms: PharmMapper (http://www.lilab-ecust.cn/pharmmapper/) (21), SwissTargetPrediction (http://www.swisstargetprediction.ch/), and DrugBank (https://www.drugbank.com) (22) to identify putative targets of fluconazole. Targets acquired from each database were consolidated, duplicate entries were removed, and all target names were standardized using the UniProt database (https://www.uniprot.org/).

### Identification of HT-related genes

To identify HT-associated genes, we systematically queried ClinVar (https://www.ncbi.nlm.nih.gov/clinvar/), GeneCards (https://www.genecards.org/) (23), and the Monarch Initiative databases (https://monarchinitiative.org/) (24) using the key terms: “liver injury,” “liver cancer,” “cirrhosis,” “hepatitis,” “cholestatic hepatitis,” and “fatty liver disease.” Results were filtered using the following criteria:

- In ClinVar, only genes with documented pathogenic clinical significance were retained;
- In GeneCards, genes were ranked by relevance score with the top 4,000 entries selected for analysis.
- In Monarch Initiative, HT-related terms were retrieved.

### Target identification and network construction for fluconazole-induced liver injury

The Venn plot of fluconazole targets and HT genes was constructed using the ggvenn package. The intersecting target proteins were then submitted to the STRING database (https://string-db.org/) for protein-protein interaction (PPI) network prediction. The resulting network file in TSV format was subsequently imported into Cytoscape 3.7.2 (http://cytoscape.org/) for visualization and analysis. Using the cytoHubba plugin within Cytoscape, we identified the top 10 hub genes through four distinct centrality algorithms: Degree, Edge Percolated Component (EPC), Maximal Clique Centrality (MCC), and Neighborhood Component Centrality (MNC).

### Enrichment analysis

Standardized gene symbol conversion and functional annotation of the target gene set were performed using the org.Hs.eg.db R package. Gene Ontology (GO) enrichment analysis across biological process (BP), molecular function (MF), and cellular component (CC) ontologies was conducted via the clusterProfiler package, while Kyoto Encyclopedia of Genes and Genomes (KEGG) pathway enrichment analysis was implemented using the DOSE package, with a significance threshold of *P* < 0.05. Visualization of GO enrichment results was achieved through bar plots generated by enrichplot, while chord diagrams representing KEGG pathway enrichments were created using the GOplot package.

### Human Protein Atlas (HPA) data collection

To confirm the hepatic relevance of the identified targets, their expression profiles in human liver tissue were retrieved from the HPA database (https://www.proteinatlas.org/). The HPA provides a comprehensive, publicly available resource of tissue- and cell-type-specific expression data based on both transcriptomics and proteomics. We queried the database for the following genes: AKT1, EGFR, ERBB2, JAK1, JAK2, KDR, and PIK3CA. The expression data for normal liver tissue, presented as normalized Transcripts Per Million (nTPM), and for specific hepatic cell types (hepatocytes, liver sinusoidal endothelial cells [LSECs], hepatic stellate cells [HSCs], Kupffer cells, T cells, B cells, plasma cells), presented as normalized Copies Per Million (nCPM), were extracted directly from the HPA portal.

### Gene Expression Omnibus (GEO) data collection

The GEO database (https://www.ncbi.nlm.nih.gov/geo/) represents a global public repository for high-throughput genomic data sets. We systematically queried GEO using the key terms “cholestatic liver injury” and “cholestasis.” Following rigorous screening, two bulk RNA-seq and one snRNA-seq data sets meeting inclusion criteria were selected for downstream analysis:

- The extrahepatic cholestatic liver injury data set GSE183754, comprising liver tissue specimens from four patients with obstructive cholestasis versus three matched controls;
- The intrahepatic cholestatic liver injury data set GSE38974, featuring *in vitro* hepatocyte models of cholestatic injury (three atazanavir, three cyclosporin A, and three nefazodone-induced cholestatic liver injury) with three control replicates;
- The single-cell transcriptional data GSE237622, including nine liver samples obtained from seven patients diagnosed with cholestatic liver injury.

### Differential expression analysis

To explore the expression of our predicted targets in general cholestasis models, differential expression analysis was performed using the limma package. Differentially expressed genes (DEGs) were identified using the following thresholds: |log₂(fold change)| > 1 and *P* value < 0.05. Visualization included volcano plots generated with ggplot2 and hierarchical clustering heatmaps created with pheatmap package using z-score-normalized expression of significant DEGs.

### Single-cell RNA sequencing analysis

We implemented stringent quality control criteria for single-cell RNA-seq data reliability. Cells were retained if they exhibited mitochondrial gene content below 25% and contained between 200 and 6,000 detected genes, with genes requiring expression in at least three cells. A total of 47,688 high-quality cells were selected for downstream analysis. To correct batch effects and enhance clustering precision, the Harmony algorithm was employed for data integration. Gene expression data were normalized using log-normalization, and the top 2,000 highly variable genes were identified via the FindVariableFeatures function. Principal component analysis (PCA) was performed for dimensionality reduction, followed by soft k-means clustering using the Harmony package (25). Cell clusters were subsequently defined using the FindClusters function at a resolution parameter of 0.2. Cell type annotation was performed based on canonical marker genes, differential expression patterns, and established cell lineages (26). Expression distribution of identified fluconazole-associated HT genes across identified cell types was further analyzed to elucidate potential cellular mechanisms underlying fluconazole-induced liver injury.

### Molecular docking

To investigate potential binding interactions between fluconazole and the potential core targets AKT1, ERBB2, and KDR, we performed molecular docking simulations. Three-dimensional protein structures were retrieved from the RCSB Protein Data Bank (https://www.rcsb.org/) using the following crystallographic entries: AKT1 (PDB ID: 1H10), ERBB2 (PDB ID: 1MFG), and KDR (PDB ID: 1VR2). Protein structures were preprocessed using PyMOL v2.3.0 (the PyMOL Molecular Graphics System, Schrödinger, LLC) by removing crystallographic water molecules and native ligands. Molecular docking simulations were conducted via the CB-Dock2 web server (https://cadd.labshare.cn/cb-dock2/) to predict binding poses and affinities of fluconazole against these targets (27). Resultant docking complexes were visualized and analyzed in PyMOL v2.3.0 to characterize specific fluconazole-protein interactions, including hydrogen bonding, hydrophobic contacts, and electrostatic potentials.

### Statistical analysis of data

Normality of continuous variables was assessed using the Shapiro-Wilk test. Normally distributed data were expressed as mean ± standard deviation (SD) and analyzed with Student’s *t*-test, while non-normally distributed variables were reported as median (first quartile [Q1], third quartile [Q3]) and compared using Mann-Whitney *U* test comparisons. Categorical variables were summarized as frequency (%) and assessed using either χ or Fisher’s exact test. *P* values resulting from all univariate comparisons between HT and non-HT groups were adjusted for multiple testing using the Benjamini-Hochberg false discovery rate (FDR) method. Clinically relevant variables and those showing univariate associations (*P* < 0.100) underwent multivariate modified Poisson regression analysis with robust error variance.

The predictive accuracy of hepatic biomarkers for fluconazole-induced liver injury in PC patients was assessed using receiver operating characteristic (ROC) curve analysis, with the area under the curve (AUC) to compare discriminatory performance across biomarkers. Optimal cutoff values for hepatic biomarkers were determined by maximizing the Youden index (sensitivity + specificity − 1) in the ROC analysis. Bootstrap resampling was performed to calculate 95% confidence intervals using the percentile method. The multivariable predictive models were constructed using logistic regression.

All statistical analyses were performed using R (v4.4.2, R Foundation). Statistical significance was defined as a two-tailed *P* < 0.05.

## RESULTS

### Patient selection and classification

After selection, there are 139 patients with PC receiving fluconazole treatment, of whom 123 demonstrated preserved baseline hepatic function (serum ALT/AST ≤ 40 U/L) and 16 exhibited pre-existing hepatic impairment. Patients with intact baseline liver function were subsequently stratified according to immune function and tertiles of baseline hepatic function markers (Fig. 2).

### Demographics and clinical characteristics

The cohort included 123 patients with PC (male: 73 cases [59.3%], female: 50 cases [40.7%]), with a mean age of 49.8 ± 13.3 years old. Comorbidity analysis revealed hypertension prevalence in 26.0% (32/123) of participants. Cough (61 cases [49.6%]), expectoration (38 [30.9%]), and fever (31 [25.2%]) constituted the most frequent symptoms.

### HT in fluconazole-treated patients

During follow-up, 29 (23.6%), 11 (8.9%), 1 (0.8%), and 5 (4.1%) patients met CTCAE, CIOMS, DILIN, and DEWG criteria, respectively. No significant association was found between fluconazole dosage and HT incidence (*P* > 0.05) (Table 1). The median time to onset of fluconazole-associated HT was 2 months (IQR 0.5–3.0 months). The patients who developed liver injury demonstrated significantly elevated baseline liver enzymes: ALT (median 23 vs 16 U/L, FDR-adj. *P* = 0.048) and GGT (30 vs 23 U/L, FDR-adj. *P* = 0.048). As shown in Table 1, immunocompromised status was more prevalent in the HT group, showing a trend toward statistical significance (44.8% vs 19.2%, FDR-adj. *P* = 0.053). Although neutropenia showed a trend toward higher prevalence in the HT group (10.3% vs 0%), this difference did not reach statistical significance (FDR-adj. *P* = 0.093) (Table 3). Neutropenia in all three cases in the HT group was related to chemotherapy diffuse large B-cell lymphoma, chronic hepatitis B, and myelodysplastic syndromes. Although autoimmune diseases showed a trend toward higher prevalence in the HT group (27.6% vs 12.8%), this difference did not reach statistical significance (*P* = 0.109) (Table 2). Comparisons of immune function and different tertile levels of hepatic biomarkers among the PC patients are presented in Tables S1 to S7.

**TABLE 1:** Patients with fluconazole-associated liver injury diagnosed by four different criteria^*a*^

Criteria	Total (*n* = 123)	Fluconazole <400 mg/day (*n* = 16)	Fluconazole = 400 mg/day (*n* = 98)	Fluconazole >400 mg/day (*n* = 9)
CTCAE	29 (23.60)	2 (12.50)	26 (26.53)	1 (11.11)
CIOMS	11 (8.94)	2 (12.50)	9 (9.18)	0 (0)
DILIN	1 (0.81)	1 (6.25)	0 (0)	0 (0)
DEWG	5 (4.07)	1 (6.25)	4 (4.08)	0 (0)

^*a*^ CTCAE, Common Terminology Criteria for Adverse Events; CIOMS, Council for International Organizations of Medical Sciences; DILIN, Drug-Induced Liver Injury Network; DEWG, Drug-Induced Liver Injury Expert Working Group.

**TABLE 2:** The baseline information of the PC patients in this study^*b*^

Variables	Total (*N* = 123)	HT group (*N* = 29)	Non-HT group (*N* = 94)	*P* value	FDR-adj. *P*
Age (years), mean ± SD	49.80 ± 13.28	50.72 ± 13.88	49.51 ± 13.16	0.669	0.928
Male/female, *n* (%)	73 (59.35)	19 (65.52)	54 (57.45)	0.439	0.856
Antibiotic use, *n* (%)	61 (49.59)	11 (37.93)	50 (53.19)	0.151	0.439
History of operation, *n* (%)	37 (30.08)	11 (37.93)	26 (27.66)	0.292	0.667
Smoking, *n* (%)	17 (13.82)	3 (10.34)	14 (14.89)	0.754	0.928
Comorbidity, *n* (%)					
Hypertension	32 (26.02)	6 (20.69)	26 (27.66)	0.455	0.856
Diabetes mellitus	18 (14.63)	5 (17.24)	13 (13.83)	0.878	1.000
Renal insufficiency	9 (7.32)	2 (6.90)	7 (7.45)	1.000	1.000
Autoimmune diseases	20 (16.26)	8 (27.59)	12 (12.77)	0.109	0.423
Connective tissue disease	5 (4.07)	1 (3.45)	4 (4.26)	1.000	1.000
Glomerulonephritis	3 (2.44)	2 (6.90)	1 (1.06)	0.138	0.439
Autoimmune thyroid disease	8 (6.50)	3 (10.34)	5 (5.32)	0.597	0.910
Clinical symptoms at onset, *n* (%)					
Cough	61 (49.59)	15 (51.72)	46 (48.94)	0.793	0.940
Expectoration	38 (30.89)	11 (37.93)	27 (28.72)	0.348	0.742
Hemoptysis	8 (6.50)	3 (10.34)	5 (5.32)	0.597	0.910
Chest distress	11 (8.94)	0 (0.00)	11 (11.70)	0.119	0.423
Chest pain	10 (8.13)	3 (10.34)	7 (7.45)	0.912	1.000
Fatigue	8 (6.50)	0 (0.00)	8 (8.51)	0.232	0.571
Fever	31 (25.20)	6 (20.69)	25 (26.60)	0.522	0.910
Liver function test, median (Q1, Q3)					
TB (μmol/L)	8.30 (5.90, 11.60)	8.60 (6.50, 13.90)	8.20 (5.82, 10.97)	0.644	0.928
DB (μmol/L)	2.90 (1.80, 4.10)	2.50 (1.70, 4.30)	3.00 (1.83, 4.10)	0.714	0.928
ALT (U/L)	18.00 (13.00, 24.00)	23.00 (18.00, 32.00)	16.00 (12.25, 23.00)	0.002^*a*^	0.048^*a*^
AST (U/L)	19.00 (15.00, 22.50)	21.00 (16.00, 23.00)	18.00 (14.00, 21.00)	0.041^*a*^	0.262
GGT (U/L)	26.00 (18.00, 41.00)	30.00 (26.00, 56.00)	23.00 (18.00, 36.00)	0.003^*a*^	0.048^*a*^
ALP (U/L)	70.00 (58.50, 83.50)	72.00 (67.00, 91.00)	68.50 (55.25, 82.00)	0.055	0.293
Immunocompromised status, *n* (%)	31 (25.20)	13 (44.83)	18 (19.15)	0.005^*a*^	0.053
Glucocorticoid use	12 (9.76)	5 (17.24)	7 (7.45)	0.232	0.571
Use of immunosuppressant	12 (9.76)	3 (10.34)	9 (9.57)	1.000	1.000
Chemotherapy	5 (4.07)	2 (6.90)	3 (3.19)	0.730	0.928
Autoimmune diseases	20 (16.26)	8 (27.59)	12 (12.77)	0.109	0.423
Immunodeficiency diseases	3 (2.44)	1 (3.45)	2 (2.13)	0.557	0.910
Neutropenia	3 (2.44)	3 (10.34)	0 (0.00)	0.012^*a*^	0.096

^*a*^ Indicate significant differences.

^*b*^ FDR, false discovery rate; SD, standard deviation; HT, hepatotoxicity; TB, total bilirubin; DB, direct bilirubin; ALT, alanine aminotransferase; AST, aspartate aminotransferase; GGT, gamma-glutamyl transpeptidase; ALP, alkaline phosphatase; TB, total bilirubin; DB, direct bilirubin.

**TABLE 3:** Treatment of PC patients^*b*^

Variables	Total (*N* = 123)	HT group (*N* = 29)	Non-HT group (*N* = 94)	*P* value	FDR-adj. *P*
Concomitant medications, *n* (%)	69 (56.10)	23 (79.31)	46 (48.94)	0.004^*a*^	0.056
ACEI/ARB	18 (14.63)	5 (17.24)	13 (13.83)	0.878	0.933
Calcium antagonists	15 (12.20)	2 (6.90)	13 (13.83)	0.501	0.933
Diuretics	4 (3.25)	2 (6.90)	2 (2.13)	0.236	0.661
Oral hypoglycemic drugs	13 (10.57)	4 (13.79)	9 (9.57)	0.764	0.933
Insulin	5 (4.07)	2 (6.90)	3 (3.19)	0.730	0.933
Statins	6 (4.88)	2 (6.90)	4 (4.26)	0.933	0.933
Anti-tuberculosis drugs	3 (2.44)	2 (6.90)	1 (1.06)	0.138	0.494
Glucocorticoid	12 (9.76)	5 (17.24)	7 (7.45)	0.232	0.571
Immunosuppressants	12 (9.76)	3 (10.34)	9 (9.57)	1.000	1.000
Antifungal treatment					
Initial dose of fluconazole (mg/day), mean ± SD	395.53 ± 76.82	398.28 ± 54.25	394.68 ± 82.78	0.827	0.933
Dose adjustments, *n* (%)	14 (11.38)	6 (20.69)	8 (8.51)	0.141	0.494
Increase	7 (5.69)	3 (10.34)	4 (4.26)	0.436	0.933
Decrease	4 (3.25)	0 (0.00)	4 (4.26)	0.572	0.933
Discontinuation	3 (2.44)	3 (10.34)	0 (0.00)	0.012^*a*^	0.084
Course of treatment, median (Q₁, Q₃)	7.00 (4.00, 9.00)	6.00 (4.00, 10.00)	7.50 (5.00, 8.75)	0.762	0.933

^*a*^ Indicate significant differences.

^*b*^ FDR, false discovery rate; SD, standard deviation; HT, hepatotoxicity; ACEI/ARB, angiotensin-converting enzyme inhibitor/angiotensin receptor blocker.

### Fluconazole treatment

All patients received fluconazole therapy at 200–600 mg/day, with dose adjustments occurring in 14 cases (11.4%): seven required escalations for disease progression, four were reduced due to adverse effects, and three were discontinued because of HT. The median treatment duration was 7 months. Concomitant medication use (glucocorticoid, immunosuppressants, antihypertensives, antidiabetics, lipid-lowering agents, and antituberculosis drugs) was more frequent in the HT group but did not meet statistical significance (79.3% vs 48.9%, FDR-adj. *P* = 0.056). A higher discontinuation rate was observed in patients with HT, with symptoms approaching significance (10.3% vs 0%; FDR-adj. *P* = 0.084), while other treatment parameters—including initial dose, dose modification frequency, and duration—showed no intergroup differences (all FDR-adj. *P* > 0.400) (Table 3).

### Characteristics of fluconazole-induced liver injury

Among the 29 patients with fluconazole-associated HT, the analysis revealed the following pattern distribution: cholestatic injury predominated (*n* = 16, 55.2%), followed by mixed injury (*n* = 11, 37.9%), with hepatocellular injury representing the minority phenotype (*n* = 2, 6.9%).

**Fig 3:**
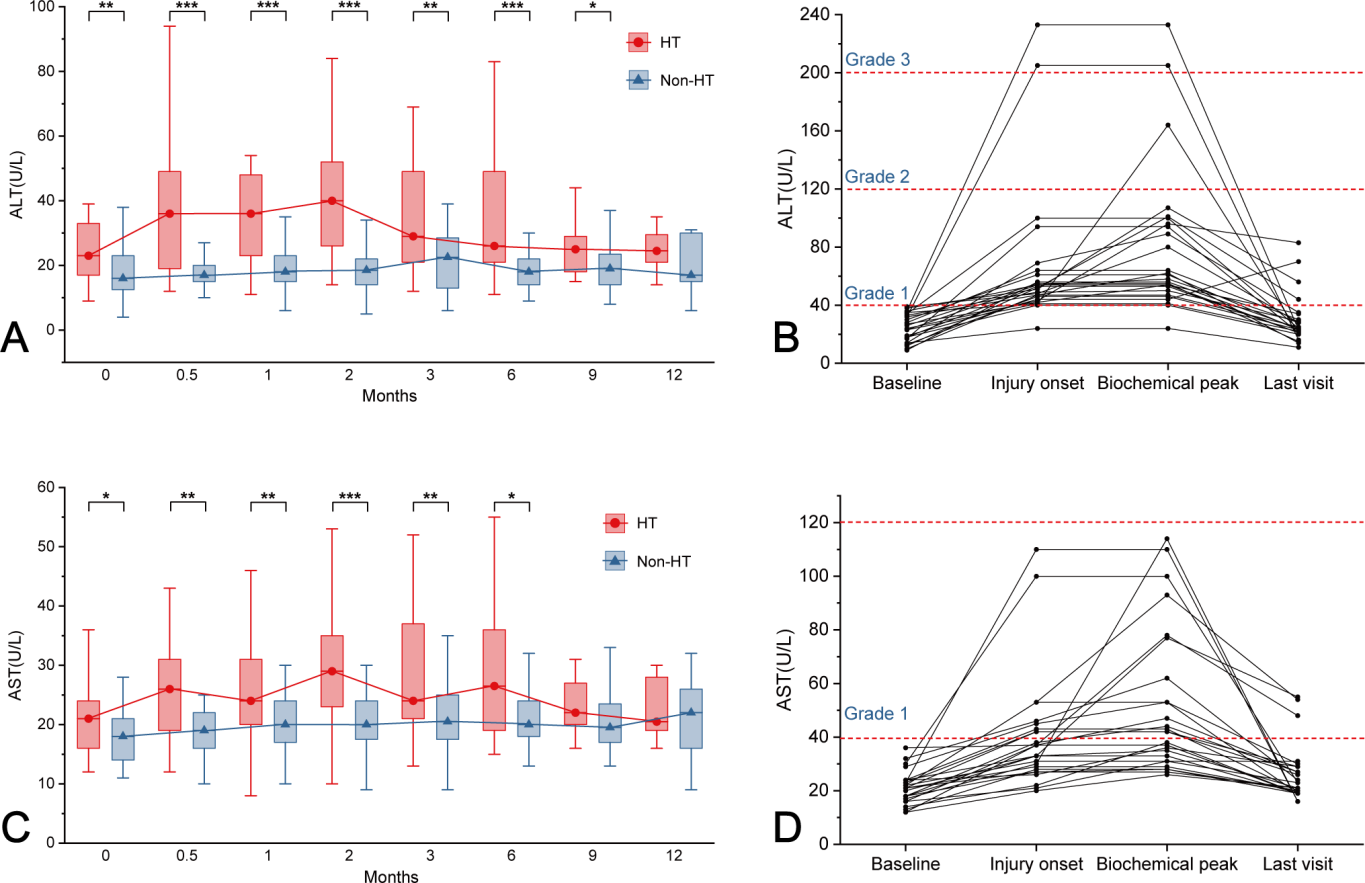
HT in fluconazole-treated patients. (**A**) Changes in serum ALT of fluconazole-treated patients during follow-up. (**B**) Serum ALT of patients in the HT group at four clinical phases. (**C**) Changes in serum AST of fluconazole-treated patients during follow-up. (**D**) Serum AST of patients in the HT group at four clinical phases. ALT, alanine aminotransferase; AST, aspartate aminotransferase; HT, hepatotoxicity.

At the last visit, 26 of 29 patients who developed fluconazole-associated HT underwent reassessment of liver function tests. Among the 26 patients, HT severity was distributed as CTCAE Grade 1 in 88.5% (23/26), Grade 2 in 3.8% (1/26), and Grade 3 in 7.7% (2/26). During follow-up, 38.5% (10/26) of patients with HT exhibited subsequent elevation in ALT levels, while 53.8% (14/26) demonstrated progressive increase in AST levels post-injury. Normalization occurred in 84.6% (22/26) for ALT and 88.5% (23/26) for AST at final follow-up. Notably, 15.4% (4/26) exhibited paradoxical enzyme elevation persisting at Grade 1 (Fig. 3B and D). Longitudinal analysis revealed significantly higher hepatic enzymes in the HT group at the first 9 months of follow-up (all *P* < 0.05), with convergence by 12-month follow-up (ALT 24.5 vs 17.0 U/L, *P* > 0.05; AST 20.5 vs 22 U/L, *P* > 0.5). Moreover, liver enzyme levels in patients with HT peaked during the 2 months of follow-up (Fig. 3A and C).

### Fluconazole-associated HT in immunocompromised patients

**Fig 4:**
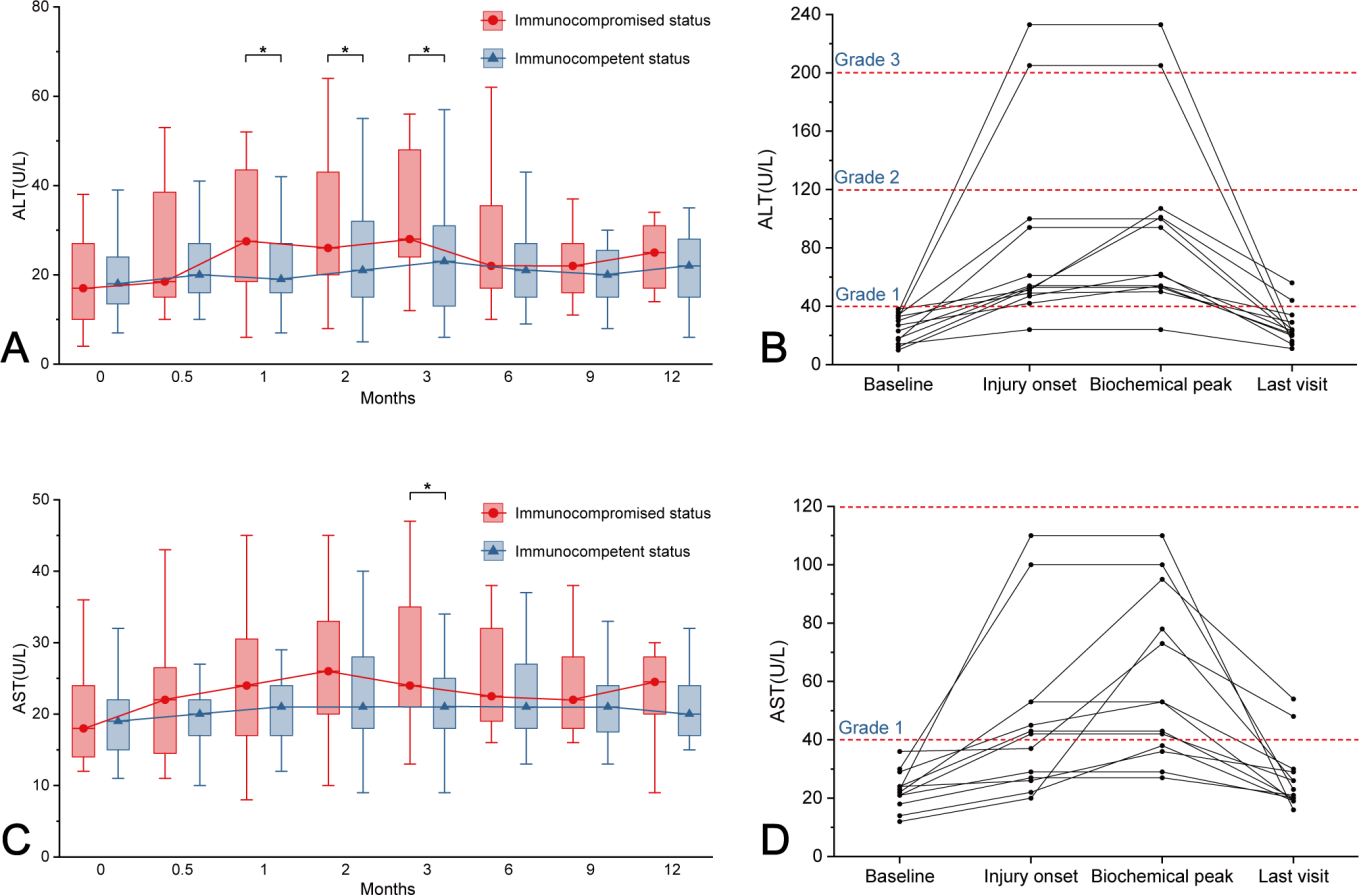
Fluconazole-associated HT in immunocompromised individuals. (**A**) Changes in serum ALT of immunocompromised patients during follow-up. (**B**) Serum ALT of immunocompromised HT patients at four clinical phases. (**C**) Changes in serum AST of immunocompromised patients during follow-up. (**D**) Serum AST of immunocompromised HT patients at four clinical phases. ALT, alanine aminotransferase; AST, aspartate aminotransferase; HT, hepatotoxicity.

Among the 31 immunocompromised patients, 13 developed HT during follow-up, with 15.4% (2/13) in CTCAE Grade 3 and 84.6% (11/13) in CTCAE Grade 1. Persistent hepatic dysfunction was observed in 15.4% (2/13) of patients at the final follow-up assessment (Fig. 4B and D). Longitudinal monitoring demonstrated persistently elevated ALT levels in immunocompromised patients relative to immunocompetent counterparts during fluconazole therapy, with peak differences observed at month 1 (27.5 vs 19.0 U/L; *P* = 0.032), sustained elevation at month 2 (26.0 vs 21.0 U/L; *P* = 0.040), and progressive divergence by month 3 (28.0 vs 23.0 U/L; *P* = 0.013). Notably, AST levels exhibited delayed intergroup disparity, achieving statistical significance exclusively at the third-month follow-up (24.0 vs 21.0 U/L; *P* = 0.032) (Fig. 4A and C).

### Effect of immunocompromised status on fluconazole-associated HT

Multivariate modified Poisson regression incorporating clinically relevant variables (DB, renal failure, and age) revealed significant associations between baseline immunocompromised status and fluconazole-associated HT (adjusted relative risk [aRR] 2.67, 95% CI 1.37–5.20; *P* = 0.004 (Table 4).

**TABLE 4:** Multivariate modified Poisson regression showing the association between immunocompromised status and fluconazole-associated hepatotoxicity

Variables	aRR (95% CI)	*P*.adj
Immunocompromised status	2.67 (1.37–5.20)	0.004^*a*^
DB (μmol/L)	1.02 (0.86–1.20)	0.858
Renal insufficiency	0.62 (0.18–2.07)	0.436
Age (years)	1.00 (0.97–1.02)	0.785

^*a*^ Indicate significant differences.

### Association between hepatic biomarkers and fluconazole-associated HT

**Fig 5:**
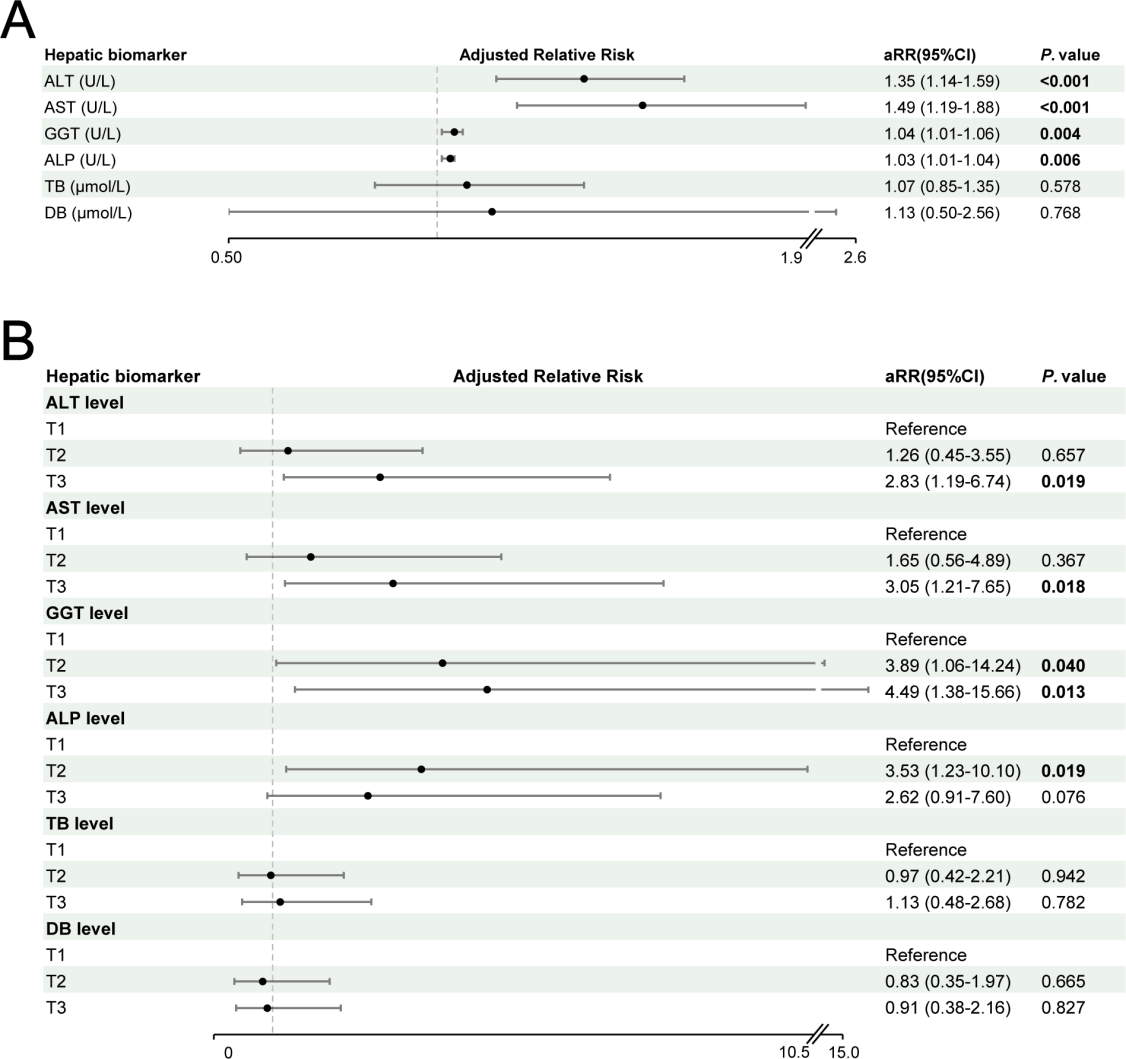
The forest plots show the association between hepatic biomarkers and fluconazole-associated HT. (**A**) Hepatic biomarker as continuous variable. (**B**) Tertiles of hepatic biomarker. HT, hepatotoxicity.

After multivariable adjustment, baseline hepatic function markers retained significant predictive value for fluconazole-associated HT. In continuous variable analysis, elevated baseline levels demonstrated concentration-dependent associations: ALT (aRR 1.35 per 5 U/L, 95% CI 1.14–1.59; *P* < 0.001), AST (aRR 1.49 per 5 U/L, 95% CI 1.19–1.88; *P* < 0.001), GGT (aRR 1.04 per 5 U/L, 95% CI 1.01–1.06; *P* = 0.004), and ALP (aRR 1.03 per 5 U/L, 95% CI 1.01–1.04; *P* = 0.006) (Fig. 5A). In tertile-based analysis, the upper ALT tertile (aRR 2.83, 95% CI 1.19–6.74; *P* = 0.019) and the upper AST tertile (aRR 3.05, 95% CI 1.21–7.65; *P* = 0.018) were observed as significant predictive factors in fluconazole-associated HT. For GGT, both middle (aRR 3.89, 95% CI 1.06–14.24; *P* = 0.040) and upper tertiles (aRR 4.49, 95% CI 1.38–15.66; *P* = 0.013) demonstrated progressive risk increases. Notably, the middle ALP tertile showed significant association (aRR 3.53, 95% CI 1.23–10.10; *P* = 0.019), whereas the upper tertile exhibited marginal significance (aRR 2.62, 95% CI 0.91–7.60; *P* = 0.076) (Fig. 5B). TB and DB showed no associations both in continuous variable and tertile-based analysis (all *P* > 0.5).

### Predictive accuracy of liver function indicators with fluconazole-associated HT

**Fig 6:**
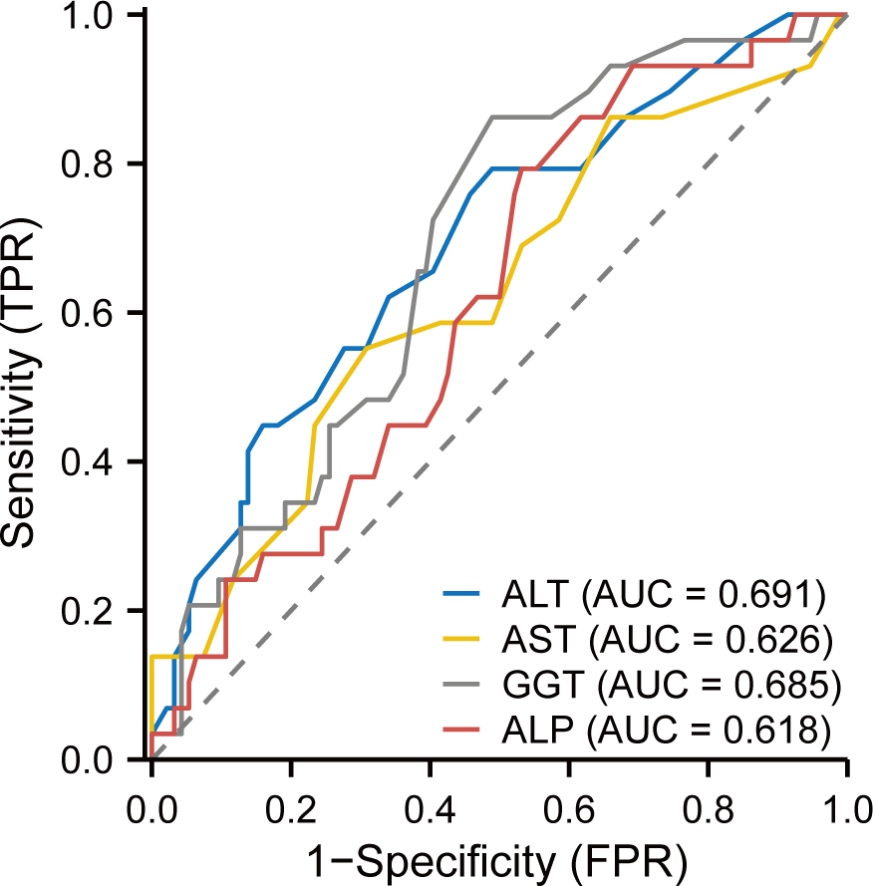
The ROC curves for exhibiting the predictive value of liver function markers with fluconazole-associated hepatotoxicity.

ROC analysis demonstrated superior predictive performance of ALT for fluconazole-associated HT in PC patients (AUC 0.69, 95% CI 0.58–0.80), followed by GGT (AUC 0.68, 95% CI 0.58–0.79), ALT (AST; AUC 0.63, 95% CI 0.50–0.75), and ALP (AUC 0.62, 95% CI 0.51–0.73) (Fig. 5). Optimal diagnostic thresholds determined by Youden index maximization were 16.5 U/L for ALT (sensitivity 51%, specificity 79%), 20.5 U/L for AST (69%, 55%), 23.5 U/L for GGT (51%, 86%), and 65.5 U/L for ALP (47%, 79%) (Table 5). The integrated multivariable models combining hepatic biomarkers with clinical factors (immunocompromised state and concomitant medications) demonstrated enhanced predictive capacity: ALT-based model (AUC 0.76, 95% CI 0.65–0.86), AST-based model (AUC 0.71, 95% CI 0.60–0.82), GGT-based model (AUC 0.74, 95% CI 0.64–0.84), ALP-based model (AUC 0.72, 95% CI 0.61–0.82), and composite model (AUC 0.76, 95% CI 0.66–0.86) (Fig. 6).

**TABLE 5:** Predictive value of hepatic biomarkers with fluconazole-associated hepatotoxicity^*a*^

	AUC (95% CI)	Accuracy (95% CI)	Sensitivity (95% CI)	Specificity (95% CI)	Cutoff
ALT (U/L)	0.69 (0.58–0.80)	0.58 (0.48–0.67)	0.51 (0.41–0.61)	0.79 (0.65–0.94)	16.5
AST (U/L)	0.63 (0.50–0.75)	0.66 (0.57–0.74)	0.69 (0.60–0.78)	0.55 (0.37–0.73)	20.5
GGT (U/L)	0.68 (0.58–0.79)	0.59 (0.50–0.68)	0.51 (0.41–0.61)	0.86 (0.74–0.99)	23.5
ALP (U/L)	0.62 (0.51–0.73)	0.54 (0.45–0.63)	0.47 (0.37–0.57)	0.79 (0.65–0.94)	65.5

^*a*^ AUC, area under the curve; ALT, alanine aminotransferase; AST, aspartate aminotransferase; GGT, gamma-glutamyl transpeptidase; ALP, alkaline phosphatase.

### Potential hepatotoxic targets of fluconazole

**Fig 7:**
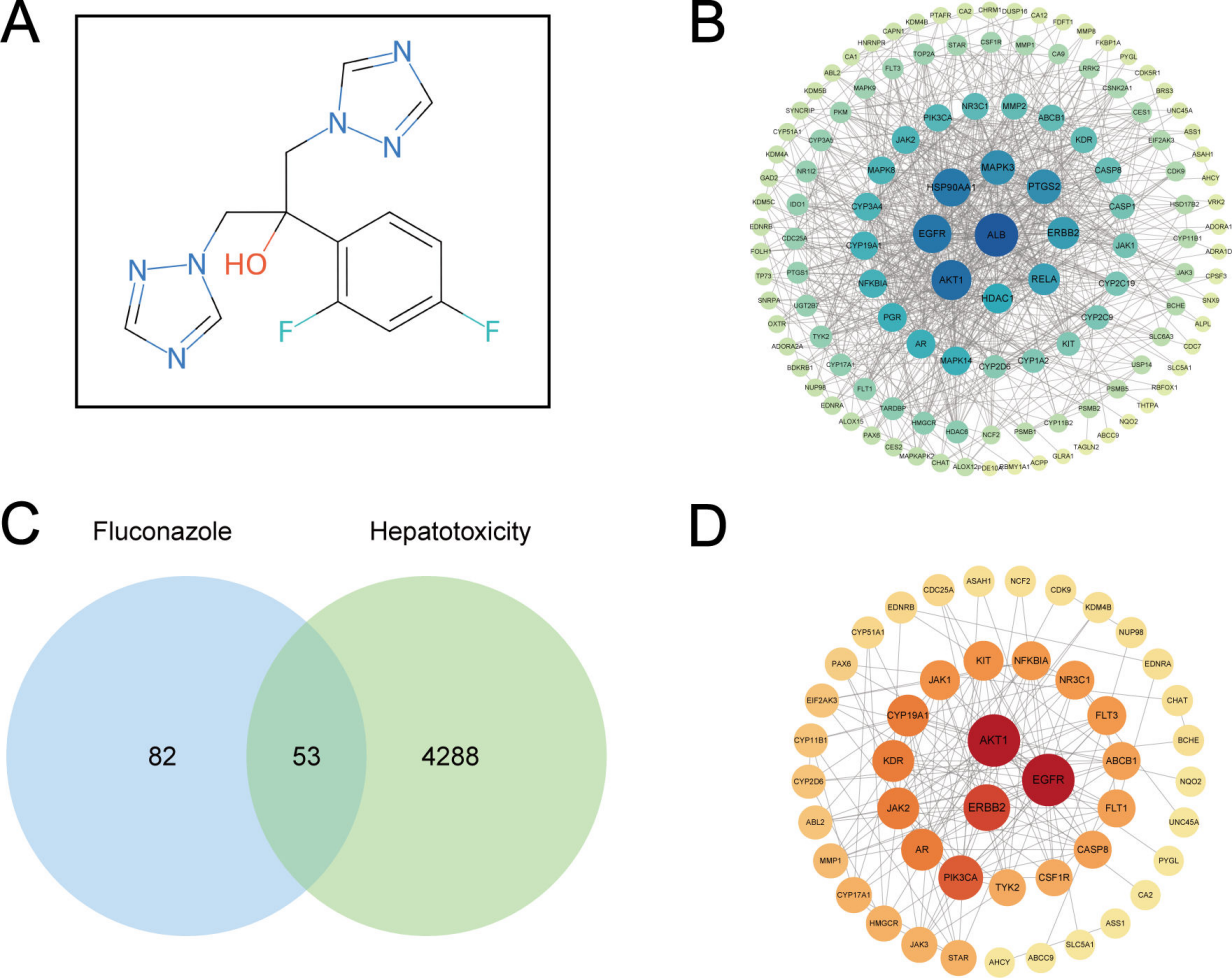
Target prediction and PPI network construction. (**A**) Chemical structure of fluconazole. (**B**) PPI network of fluconazole targets. (**C**) Venn plot of fluconazole targets and hepatotoxicity-related genes. (**D**) PPI network of hepatotoxic targets of fluconazole. PPI, protein-protein interaction; PPI, protein-protein interaction

One hundred thirty-five putative targets of fluconazole were identified through predictive analyses across four databases: DrugBank, PharmMapper, PubChem, and SwissTargetPrediction (Fig. 7B). Then, we retrieved 4,341 HT-associated genes from ClinVar, GeneCards, and Monarch Initiative resources. Venn analysis yielded 53 shared targets implicated in fluconazole-induced liver injury (Fig. 7C).

### PPI network and potential core regulators of fluconazole-associated HT

**Fig 8:**
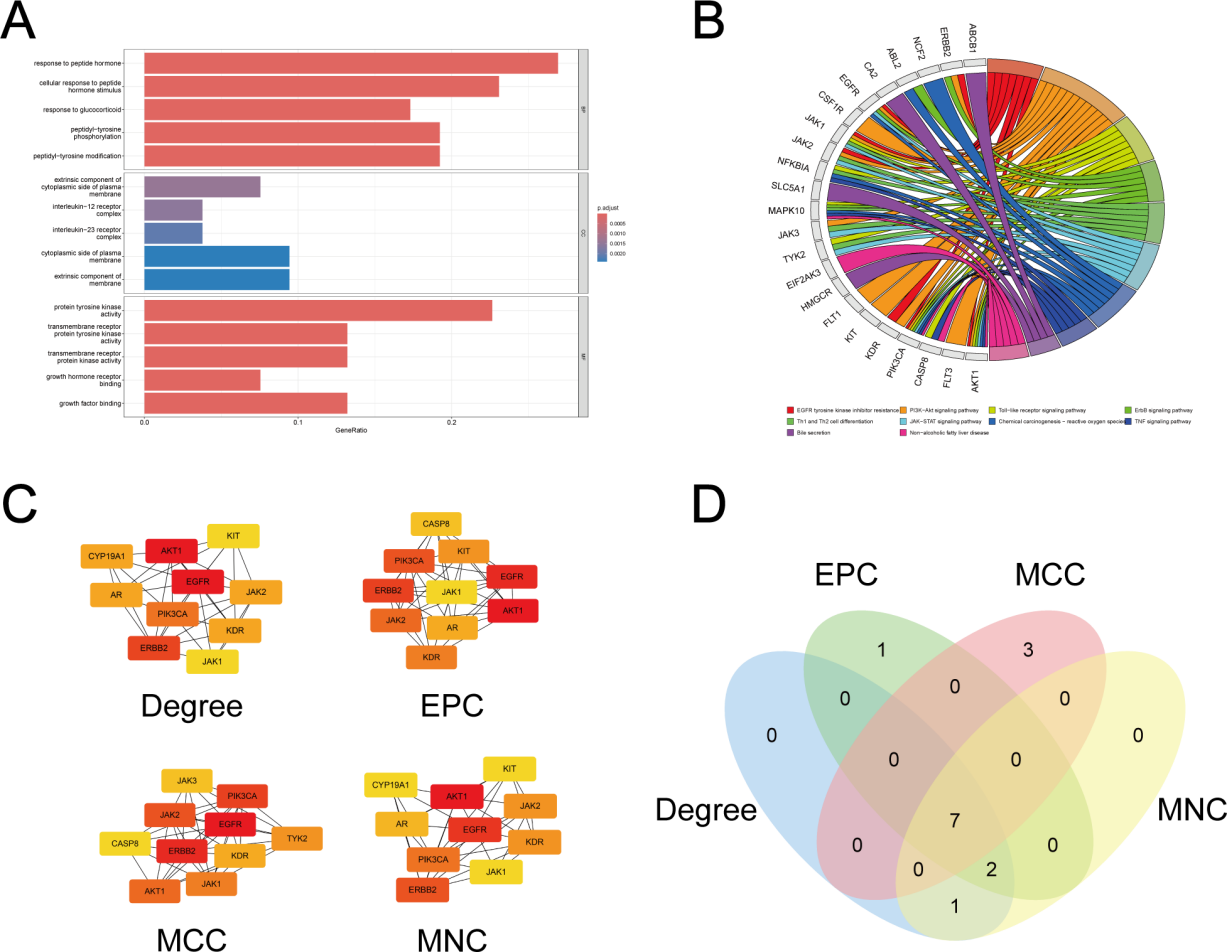
Enrichment analysis and screening of potential target genes. (**A**) GO analysis. (**B**) KEGG analysis. (**C**) Top 10 genes of four screening methods. (**D**) Venn plot of four screening methods. GO, Gene Ontology; KEGG, Kyoto Encyclopedia of Genes and Genomes; EPC, Edge Percolated Component; MCC, Maximal Clique Centrality; MNC, Neighborhood Component Centrality.

Figure 7D illustrates the PPI network encompassing targets of fluconazole-induced liver injury. Core network targets were identified through four topological algorithms: Degree, EPC, MCC, and MNC. The top 10 targets from each method were cross-referenced, yielding a consensus set of seven key regulators: AKT1, EGFR, ERBB2, JAK1, JAK2, KDR, and PIK3CA (Fig. 8C and D). Table 6 enumerates quantitative metrics for each screening parameter.

**TABLE 6:** Identified hepatotoxic genes screened from PPI network

Number	Gene	Degree	EPC	MCC	MNC
1	AKT1	24	23.716	1993	23
2	EGFR	24	23.818	3240	22
3	ERBB2	18	23.002	2622	18
4	PIK3CA	16	22.997	2460	16
5	CYP19A1	13	20.106	162	11
6	AR	13	20.914	307	12
7	JAK2	13	22.496	2112	13
8	KDR	13	21.877	892	13
9	JAK1	11	21.217	1236	11
10	KIT	11	21.228	362	11
11	NFKBIA	10	21.047	522	10
12	FLT3	10	20.238	391	9
13	NR3C1	10	20.07	65	9
14	CASP8	9	20.227	624	9
15	FLT1	9	19.35	601	8

### GO and KEGG analysis

GO enrichment analysis revealed distinct functional characteristics of fluconazole-induced liver injury targets across BP, MF, and CC ontologies. These enrichments were visualized through faceted bar plots, demonstrating significant associations with glucocorticoid response, peptide hormone response, and kinase modification processes (Fig. 8A). KEGG pathway analysis identified enrichment in tyrosine kinase signaling pathways, immune response pathways, and hepatic injury-related pathways (Fig. 8B).

### Expression of identified genes in transcriptome data

To address the potential concern regarding the liver-specific relevance of the predicted drug targets, we examined their expression in normal human liver using data from the HPA database. As summarized in Table S8, all seven core targets (AKT1, EGFR, ERBB2, JAK1, JAK2, KDR, PIK3CA) were confirmed to be expressed in normal liver tissue, with nTPM values ranging from 4.2 to 50.3. Furthermore, single-cell data revealed distinct expression patterns across major liver cell populations. Notably, KDR was predominantly expressed in liver sinusoidal endothelial cells (LSECs) (nCPM: 234.6). EGFR showed the highest expression in hepatocytes (nCPM: 298.4). JAK1 and PIK3CA were ubiquitously expressed across all queried cell types. AKT1 expression was highest in hepatocytes. ERBB2 and JAK2 displayed more varied expression, with ERBB2 being relatively higher in HSCs and JAK2 in Kupffer cells and B cells.

**Fig 9:**
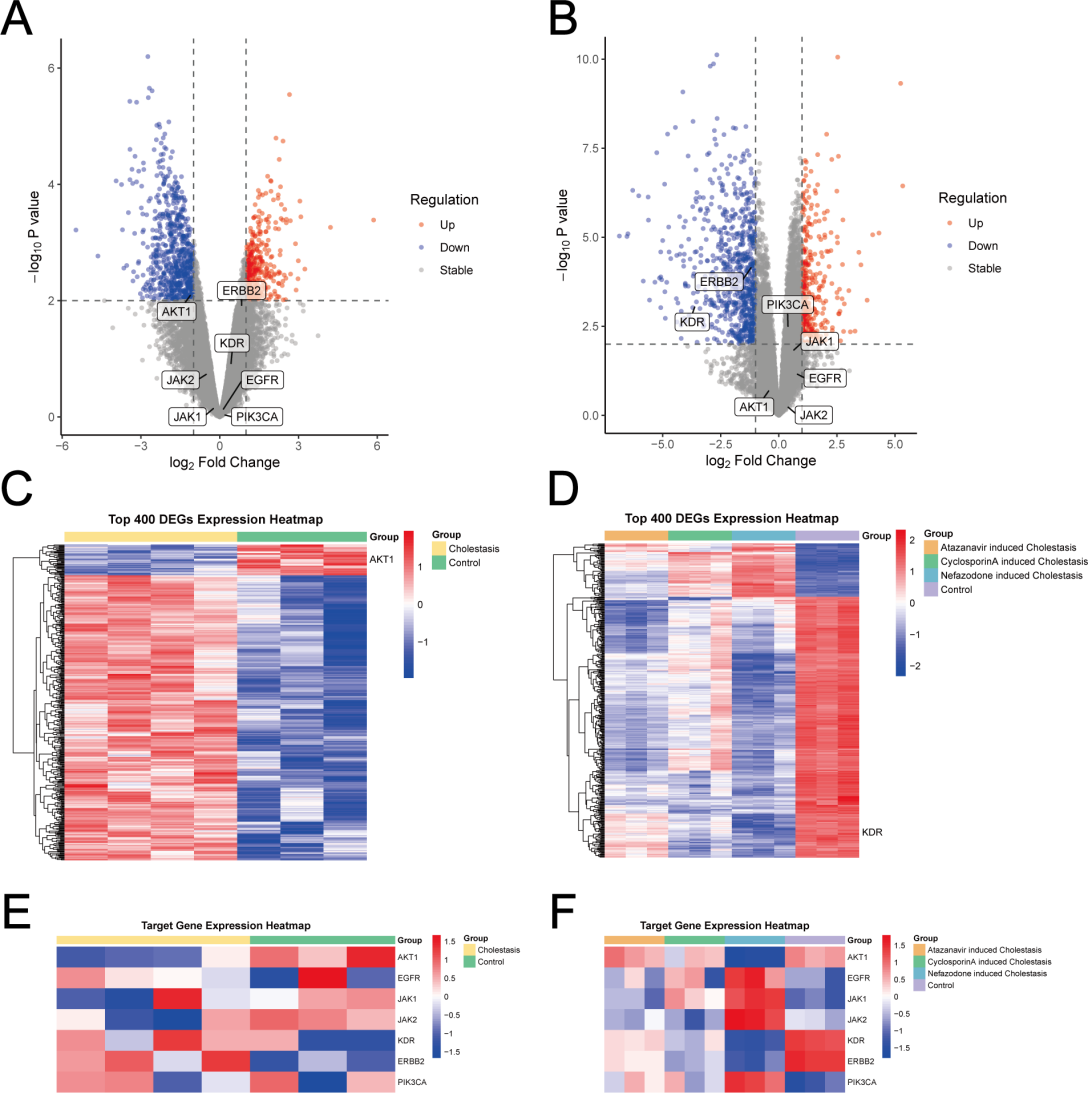
Differential expression of cholestatic liver injury. (**A**) Volcano plot of extrahepatic cholestasis. (**B**) Volcano plot of intrahepatic cholestasis. (**C**) Heatmap of DEGs in extrahepatic cholestasis. (**D**) Heatmap of DEGs in intrahepatic cholestasis. (**E**) Heatmap of identified target genes in extrahepatic cholestasis. (**F**) Heatmap of identified target genes in intrahepatic cholestasis. DEG, different expression gene.

Differential analysis of bulk RNA data revealed significant downregulation of critical target genes across cholestatic liver injury subtypes: AKT1 expression was markedly reduced in extrahepatic cholestasis (Fig. 9A, C and E), while intrahepatic cholestasis exhibited significant suppression of ERBB2 and KDR (Fig. 9B, D and F).

**Fig 10:**
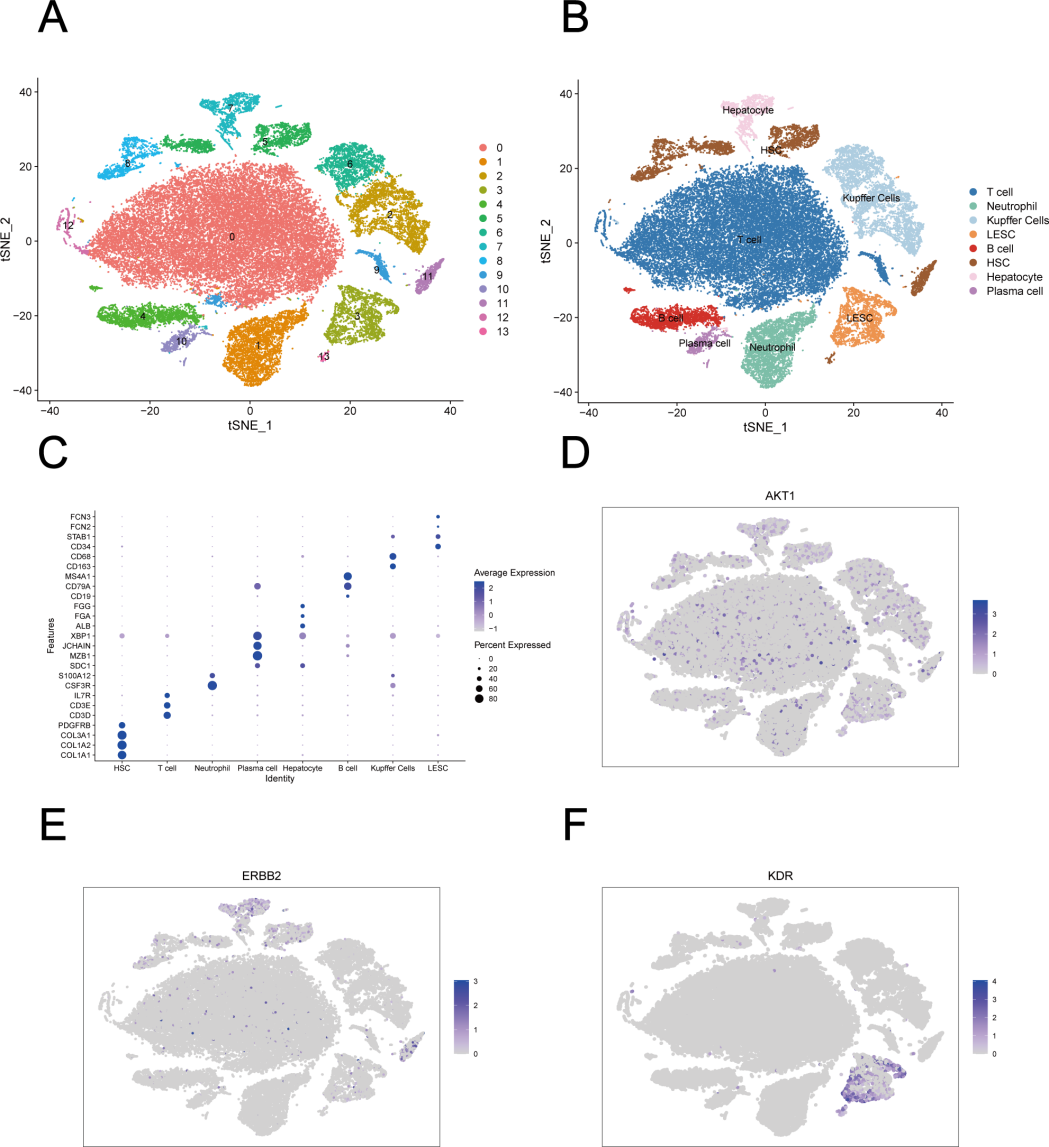
Single-cell analysis of cholestatic liver injury. (**A**) Clustering of 47,688 high-quality cells into 14 distinct clusters (resolution = 0.2). (**B**) Annotation of cell types on the basis of canonical marker genes. (**C**) Dot plot showing gene markers’ expression. (**D**) tSNE plot visualizing expression patterns of AKT1. (**E**) tSNE plot visualizing expression patterns of ERBB2. (**F**) tSNE plot visualizing expression patterns of KDR. LSEC, liver sinusoidal endothelial cell; HSC, hepatic stellate cell.

Then, single-cell analysis was performed to explore potential spatial context for fluconazole-induced HT. Following the application of the Harmony algorithm to the single-cell transcriptomic data, the cellular distribution within each sample remained largely consistent, indicating the absence of significant batch effects between samples and confirming its suitability for downstream analyses (Supplemental material). A total of 13 distinct cell clusters were identified using a resolution parameter of 0.2 (Fig. 10A). Based on canonical marker genes (Fig. 10C), we successfully annotated eight cell populations, including T cells, B cells, plasma cells, neutrophils, Kupffer cells, LSECs, HSCs, and hepatocytes (Fig. 10B). Single-cell landscape analysis revealed widespread expression of AKT1 in the liver tissues of patients with cholestatic liver injury (Fig. 10D), while ERBB2 was predominantly expressed in hepatocytes, HSCs, and T cells (Fig. 10E). Among all cell types, LSECs exhibited the highest expression levels of KDR (Fig. 10F).

### Molecular docking of potential hepatotoxic targets of fluconazole

**Fig 11:**
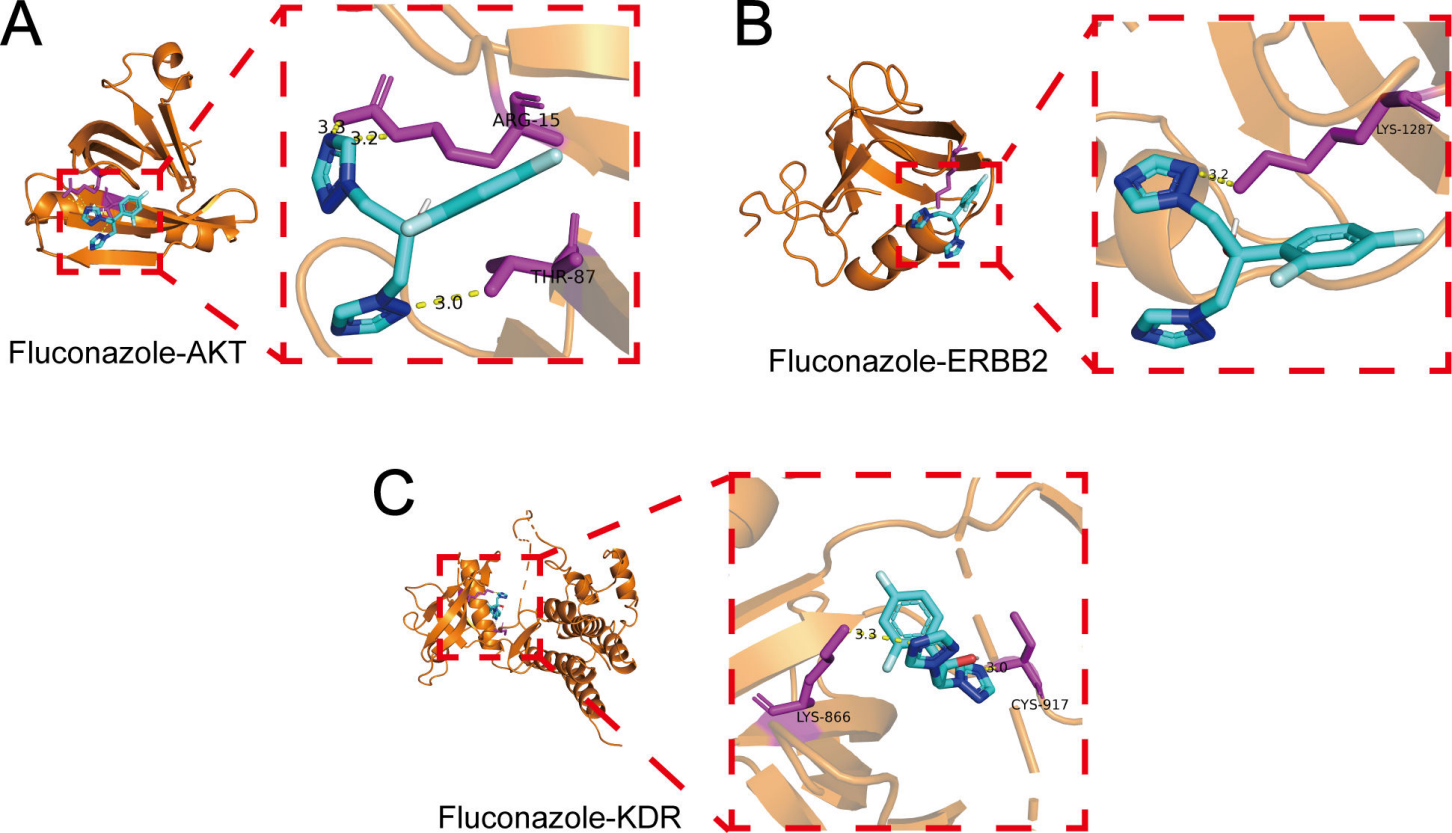
Molecular docking results with the lowest binding energy for each identified target with fluconazole. (**A**) Fluconazole with AKT. (**B**) Fluconazole with ERBB2. (**C**) Fluconazole with KDR.

Molecular docking showed significant hydrogen bonding interactions between fluconazole and the target proteins AKT1, ERBB2, and KDR, indicating strong and stable binding (Fig. 11). Fluconazole exhibited the strongest binding affinity for KDR with a binding energy of −6.5 kcal/mol—indicative of moderate-affinity interactions—while also demonstrating favorable binding to AKT1 (−5.7 kcal/mol) and ERBB2 (−5.4 kcal/mol), confirming spontaneous binding capabilities to these targets (Table 7).

**TABLE 7:** Molecular docking analysis of fluconazole and the identified genes

Ligand	Core targets	Binding energy (kcal/mol)
Fluconazole	KDR	−6.5
Fluconazole	AKT1	−5.7
Fluconazole	ERBB2	−5.4

## DISCUSSION

Monitoring of antifungal-related adverse effects constitutes a critical component in the management of PC patients. This study systematically characterizes the longitudinal hepatic alterations and HT events during fluconazole therapy, with novel exploration of the predictive value of baseline hepatic function markers and immune status for fluconazole-associated HT. Our findings confirm the working hypothesis that pre-existing hepatic enzyme elevations and immunocompromised status synergistically amplify susceptibility to fluconazole-induced liver injury in PC patients, demonstrating significant predictive utility through multivariate modeling. Additionally, the liver injury pattern was analyzed, showing that cholestasis is the dominant pattern of fluconazole-induced liver injury. Further exploration identifies AKT, ERBB2, and KDR as the potential targets of fluconazole-associated HT by integrating network toxicology, molecular docking, and transcriptomics.

A 2022 real-world study demonstrated that the incidence of voriconazole-, fluconazole-, and itraconazole-associated HT was 32.5%, 19.4%, and 14.5%, respectively (11). In our cohort, 23.6% of patients developed liver injury of CTCAE criteria during follow-up, closely aligning with these findings. Moreover, the incidence of liver injury using CIOMS, DEWG, and DILIN criteria was 0.8–8.9%. Using CTCAE criteria, we characterized hepatic dysfunction across four clinical phases (baseline, injury onset, biochemical peak, and final follow-up), revealing that the majority of PC patients exhibited mild transaminase elevations, while 10.2% developed CTCAE Grade ≥ 2 HT. Notably, 89.7% of patients continued full-dose therapy despite HT, yet 15.4% maintained elevated enzymes (>1 × ULN) at final assessment, suggesting unresolved hepatocyte stress. Additionally, longitudinal analysis revealed a biochemical peak at the 2-month follow-up for HT patients, which is in accord with the median time to onset of HT. Considering the high risk of fluconazole-induced liver injury, proactive monitoring strategies are warranted: assessment of hepatic function is required for at least 2 months, and sustained elevations of liver enzyme should trigger fluconazole dose reductions.

Immunocompromised status constitutes a key risk factor for PC, correlating with heightened symptom severity and suboptimal fluconazole responses (28). Our fluconazole-monotherapy cohort demonstrated only 25.2% (31/123) immunocompromised prevalence, starkly contrasting with the 59.6% (59/99) reported in another study (29). This discrepancy likely stems from patient selection: exclusion of patients receiving alternative/combination antifungals (19.4% of screened population) preferentially eliminated high-risk cases requiring intensive regimens (e.g., amphotericin B for CNS involvement), who are more likely to be immunocompromised individuals.

While immunocompromised individuals with PC exhibit heightened clinical severity and poorer prognoses, the effect of immunosuppression on antifungal toxicity remains underexplored. Our longitudinal data demonstrate that immunocompromised hosts developed significantly higher transaminase elevations during the first treatment trimester, with multivariate analysis revealing a 2.7-fold increased risk of HT. This novel immunopharmacological association—absent in existing azole HT models (17, 18, 30)—suggests that fluconazole-induced liver injury may involve immune-mediated pathways. The findings mandate extended hepatic monitoring in immunocompromised cohorts and provide mechanistic groundwork distinct from voriconazole/itraconazole toxicity paradigms, advocating for further immunological study on fluconazole-associated HT.

Pretreatment hepatic function constitutes a pivotal predictor of DILI risk, as evidenced by tuberculosis research demonstrating that baseline ALT elevation (per 30 U/L increment) increases antitubercular liver injury risk by 2.2-fold (31), with additional contributions from AST and TB (32). In our fluconazole safety analysis, baseline liver biomarkers exhibited dose-dependent HT associations: per 5 U/L increase, baseline ALT conferred 35% risk elevation, AST 49%, GGT 4%, and ALP 3%. Tertile stratification of liver function markers also revealed progressive risk escalation. The composite model, integrating hepatic biomarkers (optimal cutoffs: ALT 16.5 U/L; AST 20.5 U/L; GGT 23.5 U/L; ALP 65.5 U/L), immunosuppression status, and concomitant medications, achieved AUC 0.76, outperforming assessments of isolated biomarkers (AUC 0.62–0.69), but still showed limited discriminatory value. Although baseline liver enzyme elevation may reflect pre-existing subclinical liver disease rather than specific susceptibility of fluconazole-induced HT, these findings validate pretreatment liver function screening as a strategic risk-mitigation measure, consistent with pharmacovigilance principles established in antimicrobial therapies (31, 32).

Through network toxicology screening, followed by expression and binding exploration using transcriptomics and molecular docking, we identified AKT1, ERBB2, and KDR as potential regulatory targets mediating fluconazole-associated HT. Moreover, single-cell landscape analysis revealed expression of identified genes in different cell types, providing spatial context for where these potential interactions might occur. AKT1 (PKBα, protein kinase Bα), a serine/threonine kinase crucial for hepatic homeostasis (33), demonstrated significant downregulation in cholestatic liver injury within our study. Given that AKT1 deficiency induces liver injury and inflammation in mice (34), and its activation protects against diverse hepatotoxic insults via pathways, such as PI3K/AKT and AKT/Nrf2 (35, 36), this suppression suggests that fluconazole may promote cholestatic injury by reducing AKT1 levels. Similarly, expression of ERBB2 (human epidermal growth factor receptor 2, HER2), a receptor tyrosine kinase frequently overexpressed in cancer, was downregulated in cholestatic injury. Although ERBB2 inhibition is clinically effective in oncology (37, 38), HER2-targeted therapies carry a substantial HT risk (39, 40), with cellular evidence implicating internalization and lysosomal co-localization in drug-induced liver damage (41). The observed ERBB2 downregulation in transcriptomics data therefore points to its inhibition as a potential mechanism in fluconazole-induced liver injury. The relationship between the tyrosine kinase receptor KDR (vascular endothelial growth factor receptor 2, VEGFR2) and liver injury, however, appeared more complex: while some reports associate KDR signaling activation with hepatic repair post-injury (42, 43), others indicate that KDR inhibitors can attenuate injury by suppressing angiogenesis, such as in carbon tetrachloride models. Collectively, these findings elucidate AKT1 suppression and ERBB2 inhibition as key mechanisms underlying general cholestatic liver injury, which would be consistent with their potential role in fluconazole-induced cholestasis, with KDR playing a potentially multifaceted role (44).

While our study identified potential targets of fluconazole-induced HT and explored the underlying mechanism, it is important to note that HT mechanisms likely differ among various azole antifungals, which may explain the clinical observation that patients who develop liver injury on one triazole (e.g., voriconazole) can often tolerate another (e.g., isavuconazole or posaconazole) (45, 46). Previous studies have proposed distinct pathways for other azoles, such as oxidative stress linked to voriconazole and cytochrome P450 enzyme activation associated with ketoconazole and itraconazole (14–18). In contrast, our integrative analysis suggests that fluconazole-induced cholestatic injury may involve the dysregulation of signaling pathways centered on AKT1, ERBB2, and KDR. This putative mechanism, derived from network toxicology and transcriptomic data of general cholestasis models, highlights a potential specificity for fluconazole and warrants further experimental validation. The distinct mechanisms hypothesized for different azoles underscore the necessity of investigating each drug’s toxicity profile individually.

### Limitations

Our study has several methodological constraints requiring cautious interpretation. First, potential confounding effects may interfere with the conclusions of the study. Our results show that concomitant medications were significantly higher in the HT group. Upon review of the medical records, among the eight patients with autoimmune diseases in the HT group, the medications used included glucocorticoids (five cases), levothyroxine sodium (two cases), statins (one case), cyclosporine (one case), and mesalazine (one case). These drugs indeed carry potential risks of hepatotoxicity. Thus, residual confounding may interfere with our conclusion that immunosuppression is associated with fluconazole-induced HT, although the use of multivariate regression adjusted for the effects of combined medications. Moreover, although univariate analysis showed no significant association between dose adjustments and HT (20.7% vs 8.5%, *P* = 0.14), residual confounding may persist given the observational design. Future randomized controlled trials using fluconazole monotherapy and fixed-dose protocols (e.g., 400 mg/day maintenance) are needed to confirm identified risk factors.

Second, various clinical definitions of HT with different emphasis make it difficult to define fluconazole-induced liver injury. In this study, the primary clinical definition (CTCAE) used relatively low ALT/AST levels for sensitivity, but it may include mild, potentially insignificant elevations, which is consistent with the low incidence observed with more specific criteria (CIOMS, DILIN, DEWG). Injury patterns were further discussed based on the (ALT/ULN)/(ALP/ULN) ratio, and the results show that cholestatic liver injury is the dominant pattern. Nevertheless, ALT is less specific for cholestatic injury, while ALP and bilirubin are more important biomarkers. Moreover, defining and grading HT through single biochemical criteria potentially underestimate clinically significant injury. A study investigating the HT of azole antifungals revealed a notable finding: fluconazole-treated mice exhibited more pronounced hepatic histopathological alterations compared to other agents in this class, despite lacking significant elevation in serum liver enzyme levels (47). Thus, subsequent investigations should incorporate composite endpoints, including ALT, ALP, and imaging abnormalities, to improve the definition and grading of fluconazole-induced cholestatic injury.

Third, in ROC curve analysis, the discriminatory value of biomarkers (highest AUC = 0.69) is limited, and the identified cutoff (ALT >16.5 U/L), while statistically optimal from our data, is within the normal range, and its clinical utility for individual patient decision-making may be limited without further validation.

Fourth, while our cohort (*n* = 123) exceeds prior PC studies (*n* = 99) (29), the moderate sample size limits subgroup analyses for disease severity, cryptococcal burden, and the degree of adjustment for potential confounding factors in the multivariate model, particularly for rare outcomes such as liver injury defined by DILIN criteria, and leads to the insignificance of sub-items of significant items, such as immunocompromised status and concomitant medications. Multicenter prospective studies with a sufficient patient population are needed to validate the identified risk factors for liver injury.

Fifth, we did not have a standardized measure of disease severity or cryptococcal burden (e.g., fungal load) available in our retrospective cohort for adjustment. Further studies are needed to focus on adjusting for the above factors to assess fluconazole-induced liver injury more accurately.

Sixth, due to the scarcity of data on fluconazole-induced HT and the limitations of experimental conditions, analysis of downstream targets for fluconazole in this study was primarily based on public transcriptomic data of general cholestasis models. Thus, future research necessitates validation of fluconazole-treated models or patient samples, integration with genomics, proteomics, and metabolomics for comprehensive multi-omics analysis, and further *in vitro* and *in vivo* experiments to explore fluconazole-specific mechanisms.

Seventh, molecular docking used to quantify the binding between fluconazole and identified targets is a computational prediction method, which requires further experimental validation to provide direct evidence of fluconazole-induced target inhibition at clinically relevant concentrations.

These limitations notwithstanding, our comprehensive study revealed the characteristics and potential targets of fluconazole-induced liver injury in patients with PC, giving insight into future clinical and mechanistic research.

### Conclusion

Our clinical findings demonstrate that long-term surveillance should be mandated, with hepatic biomarkers requiring assessment for a minimum of 2 months, particularly in high-risk populations of fluconazole-induced HT, defined by pretreatment biomarker level exceeding established thresholds or an underlying immunocompromised status. Fluconazole-induced cholestatic injury may involve the dysregulation of signaling pathways centered on AKT1, ERBB2, and KDR, while experimental validation was required to further construct fluconazole-specific mechanisms. To sum up, our research enables risk stratification and lays a foundation for further mechanistic study on fluconazole-induced liver injury.

## Data Availability

The clinical data sets generated and/or analyzed during the current study are not publicly available due to patient privacy and confidentiality regulations but are available from the corresponding author (Hongni Jiang) upon reasonable request. The request will be subject to review and approval by the Ethics Committee of Zhongshan Hospital, Fudan University to ensure compliance with ethical standards. The transcriptomics data analyzed in this study are publicly available in the Gene Expression Omnibus (GEO) repository under accession numbers GSE183754, GSE38974, and GSE237622.

## SUPPLEMENTAL MATERIAL

Supplemental material aac.01976-25-s0001.docx Tables S1 to S8.

ASM does not own the copyrights to Supplemental Material that may be linked to, or accessed through, an article. The authors have granted ASM a non-exclusive, world-wide license to publish the Supplemental Material files. Please contact the corresponding author directly for reuse.
