# Supplementary material for: Fluconazole-induced liver injury in patients with pulmonary cryptococcosis: a comprehensive study integrating clinical cohort analysis, network toxicology, molecular docking, and transcriptomics
Source: Antimicrob Agents Chemother. 2026 Apr 20;70(6):e01976-25. doi: 10.1128/aac.01976-25 (PMC13231873; doi:10.1128/aac.01976-25)
Supplement: Supplemental material — Tables S1 to S8. [file aac.01976-25-s0001.docx]

| Variables | ALT | | | Statistic | *P* |
| --- | --- | --- | --- | --- | --- |
|  | T1 (n = 41) | T2 (n = 41) | T3 (n = 41) |  |  |
| Age(years), Mean ± SD | 51.88 ± 15.22 | 49.32 ± 12.18 | 48.20 ± 12.29 | F=0.83 | 0.441 |
| Male/female, n(%) | 20 (48.78) | 24 (58.54) | 29 (70.73) | χ²=4.11 | 0.128 |
| Initial dose of fluconazole  (mg/day), Mean ± SD | 391.46 ± 71.50 | 395.12 ± 70.54 | 400.00 ± 88.74 | F=0.13 | 0.882 |
| History of operation, n(%) | 17 (41.46) | 10 (24.39) | 10 (24.39) | χ²=3.79 | 0.150 |
| Smoking, n(%) | 5 (12.20) | 6 (14.63) | 6 (14.63) | χ²=0.14 | 0.934 |
| Antibiotic use, n(%) | 18 (43.90) | 23 (56.10) | 20 (48.78) | χ²=1.24 | 0.539 |
| **Comorbidity，n(%)** |  |  |  |  |  |
| Hypertension | 11 (26.83) | 7 (17.07) | 14 (34.15) | χ²=3.13 | 0.210 |
| Diabetes mellitus | 6 (14.63) | 6 (14.63) | 6 (14.63) | χ²=0.00 | 1.000 |
| Renal insufficiency | 4 (9.76) | 4 (9.76) | 1 (2.44) | - | 0.389 |
| Autoimmune diseases | 7 (17.07) | 6 (14.63) | 7 (17.07) | χ²=0.12 | 0.942 |
| **Clinical symptoms at onset,**  **n (%)** |  |  |  |  |  |
| Cough | 21 (51.22) | 22 (53.66) | 18 (43.90) | χ²=0.85 | 0.655 |
| Expectoration | 13 (31.71) | 13 (31.71) | 12 (29.27) | χ²=0.08 | 0.963 |
| Hemoptysis | 3 (7.32) | 1 (2.44) | 4 (9.76) | - | 0.530 |
| Chest distress | 4 (9.76) | 4 (9.76) | 3 (7.32) | - | 1.000 |
| Chest pain | 2 (4.88) | 5 (12.20) | 3 (7.32) | - | 0.602 |
| Fatigue | 3 (7.32) | 2 (4.88) | 3 (7.32) | - | 1.000 |
| Fever | 11 (26.83) | 10 (24.39) | 10 (24.39) | χ²=0.09 | 0.958 |
| Immunocompromised status | 13 (31.71) | 8 (19.51) | 10 (24.39) | χ²=1.64 | 0.441 |

**S1** Comparison of groups of different ALT level

| Variables | AST | | | Statistic | *P* |
| --- | --- | --- | --- | --- | --- |
|  | T1 (n = 41) | T2 (n = 41) | T3 (n = 41) |  |  |
| Age(years), Mean ± SD | 50.39 ± 13.75 | 47.73 ± 14.31 | 51.27 ± 11.73 | F=0.78 | 0.458 |
| Male/female, n(%) | 21 (51.22) | 23 (56.10) | 29 (70.73) | χ²=3.50 | 0.173 |
| Initial dose of fluconazole  (mg/day), Mean ± SD | 385.37 ± 64.46 | 420.73 ± 82.90 | 380.49 ± 77.36 | F=3.49 | **0.034** |
| History of operation, n(%) | 17 (41.46) | 11 (26.83) | 9 (21.95) | χ²=4.02 | 0.134 |
| Smoking, n(%) | 5 (12.20) | 3 (7.32) | 9 (21.95) | χ²=3.82 | 0.148 |
| Antibiotic use, n(%) | 21 (51.22) | 22 (53.66) | 18 (43.90) | χ²=0.85 | 0.655 |
| **Comorbidity，n(%)** |  |  |  |  |  |
| Hypertension | 9 (21.95) | 8 (19.51) | 15 (36.59) | χ²=3.63 | 0.163 |
| Diabetes mellitus | 8 (19.51) | 4 (9.76) | 6 (14.63) | χ²=1.56 | 0.458 |
| Renal insufficiency | 4 (9.76) | 2 (4.88) | 3 (7.32) | - | 0.908 |
| Autoimmune diseases | 7 (17.07) | 5 (12.20) | 8 (19.51) | χ²=0.84 | 0.658 |
| **Clinical symptoms at onset,**  **n (%)** |  |  |  |  |  |
| Cough | 21 (51.22) | 24 (58.54) | 16 (39.02) | χ²=3.19 | 0.203 |
| Expectoration | 14 (34.15) | 13 (31.71) | 11 (26.83) | χ²=0.53 | 0.766 |
| Hemoptysis | 4 (9.76) | 1 (2.44) | 3 (7.32) | - | 0.530 |
| Chest distress | 4 (9.76) | 6 (14.63) | 1 (2.44) | - | 0.181 |
| Chest pain | 2 (4.88) | 7 (17.07) | 1 (2.44) | - | 0.076 |
| Fatigue | 1 (2.44) | 4 (9.76) | 3 (7.32) | - | 0.530 |
| Fever | 12 (29.27) | 10 (24.39) | 9 (21.95) | χ²=0.60 | 0.739 |
| Immunocompromised status | 12 (29.27) | 7 (17.07) | 12 (29.27) | χ²=2.16 | 0.340 |

**S2** Comparison of groups of different AST level

| Variables | GGT | | | Statistic | *P* |
| --- | --- | --- | --- | --- | --- |
|  | T1 (n = 41) | T2 (n = 41) | T3 (n = 41) |  |  |
| Age(years), Mean ± SD | 48.39 ± 14.60 | 53.46 ± 12.10 | 47.54 ± 12.56 | F=2.44 | 0.091 |
| Male/female, n(%) | 18 (43.90) | 27 (65.85) | 28 (68.29) | χ²=6.13 | **0.047** |
| Initial dose of fluconazole  (mg/day), Mean ± SD | 391.46 ± 85.08 | 393.90 ± 66.33 | 401.22 ± 79.44 | F=0.18 | 0.838 |
| History of operation, n(%) | 11 (26.83) | 16 (39.02) | 10 (24.39) | χ²=2.40 | 0.302 |
| Smoking, n(%) | 5 (12.20) | 6 (14.63) | 6 (14.63) | χ²=0.14 | 0.934 |
| Antibiotic use, n(%) | 20 (48.78) | 23 (56.10) | 18 (43.90) | χ²=1.24 | 0.539 |
| **Comorbidity，n(%)** |  |  |  |  |  |
| Hypertension | 8 (19.51) | 14 (34.15) | 10 (24.39) | χ²=2.37 | 0.306 |
| Diabetes mellitus | 6 (14.63) | 8 (19.51) | 4 (9.76) | χ²=1.56 | 0.458 |
| Renal insufficiency | 1 (2.44) | 4 (9.76) | 4 (9.76) | - | 0.389 |
| Autoimmune diseases | 6 (14.63) | 8 (19.51) | 6 (14.63) | χ²=0.48 | 0.788 |
| **Clinical symptoms at onset,**  **n (%)** |  |  |  |  |  |
| Cough | 20 (48.78) | 20 (48.78) | 21 (51.22) | χ²=0.07 | 0.968 |
| Expectoration | 12 (29.27) | 9 (21.95) | 17 (41.46) | χ²=3.73 | 0.155 |
| Hemoptysis | 2 (4.88) | 2 (4.88) | 4 (9.76) | - | 0.729 |
| Chest distress | 3 (7.32) | 4 (9.76) | 4 (9.76) | - | 1.000 |
| Chest pain | 4 (9.76) | 1 (2.44) | 5 (12.20) | - | 0.340 |
| Fatigue | 3 (7.32) | 4 (9.76) | 1 (2.44) | - | 0.530 |
| Fever | 8 (19.51) | 12 (29.27) | 11 (26.83) | χ²=1.12 | 0.571 |
| Immunocompromised status | 7 (17.07) | 13 (31.71) | 11 (26.83) | χ²=2.42 | 0.299 |

**S3** Comparison of groups of different GGT level

| Variables | ALP | | | Statistic | *P* |
| --- | --- | --- | --- | --- | --- |
|  | T1 (n = 41) | T2 (n = 41) | T3 (n = 41) |  |  |
| Age(years), Mean ± SD | 47.61 ± 12.47 | 51.32 ± 13.49 | 50.46 ± 13.90 | F=0.87 | 0.420 |
| Male/female, n(%) | 24 (58.54) | 26 (63.41) | 23 (56.10) | χ²=0.47 | 0.790 |
| Initial dose of fluconazole  (mg/day), Mean ± SD | 391.46 ± 96.11 | 406.10 ± 65.38 | 389.02 ± 65.68 | F=0.59 | 0.557 |
| History of operation, n(%) | 9 (21.95) | 14 (34.15) | 14 (34.15) | χ²=1.93 | 0.380 |
| Smoking, n(%) | 4 (9.76) | 6 (14.63) | 7 (17.07) | χ²=0.96 | 0.620 |
| Antibiotic use, n(%) | 24 (58.54) | 19 (46.34) | 18 (43.90) | χ²=2.02 | 0.365 |
| **Comorbidity，n(%)** |  |  |  |  |  |
| Hypertension | 10 (24.39) | 11 (26.83) | 11 (26.83) | χ²=0.08 | 0.959 |
| Diabetes mellitus | 7 (17.07) | 7 (17.07) | 4 (9.76) | χ²=1.17 | 0.557 |
| Renal insufficiency | 4 (9.76) | 1 (2.44) | 4 (9.76) | - | 0.389 |
| Autoimmune diseases | 7 (17.07) | 6 (14.63) | 7 (17.07) | χ²=0.12 | 0.942 |
| **Clinical symptoms at onset,**  **n (%)** |  |  |  |  |  |
| Cough | 17 (41.46) | 20 (48.78) | 24 (58.54) | χ²=2.41 | 0.300 |
| Expectoration | 11 (26.83) | 10 (24.39) | 17 (41.46) | χ²=3.27 | 0.194 |
| Hemoptysis | 2 (4.88) | 1 (2.44) | 5 (12.20) | - | 0.272 |
| Chest distress | 4 (9.76) | 5 (12.20) | 2 (4.88) | - | 0.621 |
| Chest pain | 3 (7.32) | 4 (9.76) | 3 (7.32) | - | 1.000 |
| Fatigue | 4 (9.76) | 1 (2.44) | 3 (7.32) | - | 0.530 |
| Fever | 8 (19.51) | 12 (29.27) | 11 (26.83) | χ²=1.12 | 0.571 |
| Immunocompromised status | 8 (19.51) | 10 (24.39) | 13 (31.71) | χ²=1.64 | 0.441 |

**S4** Comparison of groups of different ALP level

| Variables | TB | | | Statistic | *P* |
| --- | --- | --- | --- | --- | --- |
|  | T1 (n = 41) | T2 (n = 41) | T3 (n = 41) |  |  |
| Age(years), Mean ± SD | 50.27 ± 13.43 | 53.37 ± 13.34 | 45.76 ± 12.25 | F=3.54 | **0.032** |
| Male/female, n(%) | 17 (41.46) | 25 (60.98) | 31 (75.61) | χ²=9.97 | **0.007** |
| Initial dose of fluconazole  (mg/day), Mean ± SD | 393.90 ± 80.00 | 387.80 ± 77.28 | 404.88 ± 74.00 | F=0.52 | 0.598 |
| History of operation, n(%) | 15 (36.59) | 14 (34.15) | 8 (19.51) | χ²=3.32 | 0.190 |
| Smoking, n(%) | 6 (14.63) | 4 (9.76) | 7 (17.07) | χ²=0.96 | 0.620 |
| Antibiotic use, n(%) | 21 (51.22) | 19 (46.34) | 21 (51.22) | χ²=0.26 | 0.878 |
| **Comorbidity，n(%)** |  |  |  |  |  |
| Hypertension | 10 (24.39) | 15 (36.59) | 7 (17.07) | χ²=4.14 | 0.126 |
| Diabetes mellitus | 7 (17.07) | 9 (21.95) | 2 (4.88) | χ²=5.08 | 0.079 |
| Renal insufficiency | 3 (7.32) | 3 (7.32) | 3 (7.32) | - | 1.000 |
| Autoimmune diseases | 10 (24.39) | 7 (17.07) | 3 (7.32) | χ²=4.42 | 0.110 |
| **Clinical symptoms at onset,**  **n (%)** |  |  |  |  |  |
| Cough | 25 (60.98) | 19 (46.34) | 17 (41.46) | χ²=3.38 | 0.184 |
| Expectoration | 14 (34.15) | 10 (24.39) | 14 (34.15) | χ²=1.22 | 0.544 |
| Hemoptysis | 4 (9.76) | 0 (0.00) | 4 (9.76) | - | 0.126 |
| Chest distress | 4 (9.76) | 2 (4.88) | 5 (12.20) | - | 0.621 |
| Chest pain | 6 (14.63) | 3 (7.32) | 1 (2.44) | - | 0.155 |
| Fatigue | 3 (7.32) | 0 (0.00) | 5 (12.20) | - | 0.096 |
| Fever | 13 (31.71) | 9 (21.95) | 9 (21.95) | χ²=1.38 | 0.502 |
| Immunocompromised status | 14 (34.15) | 11 (26.83) | 6 (14.63) | χ²=4.23 | 0.121 |

**S5** Comparison of groups of different TB level

| Variables | DB | | | Statistic | *P* |
| --- | --- | --- | --- | --- | --- |
|  | T1 (n = 41) | T2 (n = 41) | T3 (n = 41) |  |  |
| Age(years), Mean ± SD | 50.46 ± 13.40 | 52.68 ± 13.32 | 46.24 ± 12.63 | F=2.55 | 0.082 |
| Male/female, n(%) | 14 (34.15) | 27 (65.85) | 32 (78.05) | χ²=17.46 | **<.001** |
| Initial dose of fluconazole  (mg/day), Mean ± SD | 392.68 ± 49.45 | 389.02 ± 99.69 | 404.88 ± 74.00 | F=0.47 | 0.623 |
| History of operation, n(%) | 16 (39.02) | 12 (29.27) | 9 (21.95) | χ²=2.86 | 0.239 |
| Smoking, n(%) | 5 (12.20) | 4 (9.76) | 8 (19.51) | χ²=1.77 | 0.412 |
| Antibiotic use, n(%) | 21(51.22) | 17 (41.46) | 23(56.10) | χ²=1.82 | 0.402 |
| **Comorbidity，n(%)** |  |  |  |  |  |
| Hypertension | 10 (24.39) | 14 (34.15) | 8 (19.51) | χ²=2.37 | 0.306 |
| Diabetes mellitus | 6 (14.63) | 9 (21.95) | 3 (7.32) | χ²=3.51 | 0.173 |
| Renal insufficiency | 4 (9.76) | 3 (7.32) | 2 (4.88) | - | 0.908 |
| Autoimmune diseases | 11 (26.83) | 5 (12.20) | 4 (9.76) | χ²=5.13 | 0.077 |
| **Clinical symptoms at onset,**  **n (%)** |  |  |  |  |  |
| Cough | 28 (68.29) | 14 (34.15) | 19 (46.34) | χ²=9.82 | **0.007** |
| Expectoration | 17 (41.46) | 8 (19.51) | 13 (31.71) | χ²=4.65 | 0.098 |
| Hemoptysis | 4 (9.76) | 0 (0.00) | 4 (9.76) | - | 0.126 |
| Chest distress | 3 (7.32) | 3 (7.32) | 5 (12.20) | - | 0.787 |
| Chest pain | 5 (12.20) | 4 (9.76) | 1 (2.44) | - | 0.340 |
| Fatigue | 4 (9.76) | 1 (2.44) | 3 (7.32) | - | 0.530 |
| Fever | 13 (31.71) | 7 (17.07) | 11 (26.83) | χ²=2.42 | 0.299 |
| Immunocompromised status | 15 (36.59) | 9 (21.95) | 7 (17.07) | χ²=4.49 | 0.106 |

**S6** Comparison of groups of different DB level

| Variables | Immunocompromised status  (n = 31) | Immunocompetent  status  (n = 92) | Statistic | *P* |
| --- | --- | --- | --- | --- |
| Age(years), Mean ± SD | 55.81 ± 12.16 | 47.77 ± 13.09 | t=-3.01 | **0.003** |
| Male/female, n(%) | 15 (48.39) | 58 (63.04) | χ²=2.06 | 0.151 |
| Antibiotic use, n(%) | 12 (38.71) | 49 (53.26) | χ²=1.96 | 0.161 |
| History of operation, n(%) | 9 (29.03) | 28 (30.43) | χ²=0.02 | 0.883 |
| Smoking, n(%) | 3 (9.68) | 14 (15.22) | χ²=0.22 | 0.637 |
| Initial dose of fluconazole  (mg/day), Mean ± SD | 379.03 ± 60.24 | 401.09 ± 81.19 | t=1.39 | 0.168 |
| **Liver Function Test, M (Q₁, Q₃)** |  |  |  |  |
| TB (μmol/L) | 6.80 (5.25, 8.80) | 8.80 (6.30, 11.80) | Z=-1.90 | 0.057 |
| DB (μmol/L) | 2.20 (1.70, 3.20) | 3.10 (2.08, 4.23) | Z=-2.07 | **0.038** |
| ALT (U/L) | 17.00 (10.00, 25.00) | 18.00 (13.75, 24.00) | Z=-0.93 | 0.351 |
| AST (U/L) | 18.00 (14.00, 23.50) | 19.00 (15.00, 22.00) | Z=-0.25 | 0.804 |
| GGT (U/L) | 29.00 (21.50, 49.50) | 26.00 (18.00, 39.50) | Z=-1.07 | 0.286 |
| ALP (U/L) | 73.00 (62.50, 89.50) | 68.50 (54.75, 81.00) | Z=-1.88 | 0.061 |
| **Comorbidity, n(%)** |  |  |  |  |
| Hypertension | 11 (35.48) | 21 (22.83) | χ²=1.93 | 0.165 |
| Diabetes mellitus | 5 (16.13) | 13 (14.13) | χ²=0.00 | 1.000 |
| Renal insufficiency | 6 (19.35) | 3 (3.26) | χ²=6.64 | **0.010** |
| **Clinical symptoms at onset, n (%)** |  |  |  |  |
| Cough | 14 (45.16) | 47 (51.09) | χ²=0.33 | 0.568 |
| Expectoration | 8 (25.81) | 30 (32.61) | χ²=0.50 | 0.478 |
| Hemoptysis | 2 (6.45) | 6 (6.52) | χ²=0.00 | 1.000 |
| Chest distress | 1 (3.23) | 10 (10.87) | χ²=0.86 | 0.354 |
| Chest pain | 1 (3.23) | 9 (9.78) | χ²=0.60 | 0.438 |
| Fatigue | 2 (6.45) | 6 (6.52) | χ²=0.00 | 1.000 |
| Fever | 9 (29.03) | 22 (23.91) | χ²=0.32 | 0.570 |

**S7** Comparison of groups of different immune status

| **Gene expression** | AKT1 | EGFR | ERBB2 | JAK1 | JAK2 | KDR | PIK3CA |
| --- | --- | --- | --- | --- | --- | --- | --- |
| Normal liver tissue (nTPM) | 50.3 | 32.2 | 30.7 | 49.7 | 4.2 | 8.6 | 6.6 |
| Hepatocytes (nCPM) | 48.2 | 298.4 | 13.6 | 182.2 | 13.4 | 0.5 | 39.4 |
| LSECs (nCPM) | 24.9 | 38.5 | 13.2 | 370.3 | 45.5 | 234.6 | 69.8 |
| HSCs (nCPM) | 6.5 | 109.7 | 45.2 | 277.6 | 19.4 | 12.9 | 58.1 |
| Kupffer cells (nCPM) | 12.3 | 50.2 | 5.1 | 397.7 | 154.8 | 8.2 | 76.9 |
| T cells (nCPM) | 19.1 | 1.9 | 2.7 | 335.3 | 44.0 | 2.8 | 43.1 |
| B cells (nCPM) | 17.9 | 4.5 | 4.5 | 296.1 | 76.3 | 9.0 | 80.8 |
| plasma cells (nCPM) | 21.3 | 1.1 | 2.2 | 93.1 | 34.8 | 9.0 | 21.3 |

**S8** Expression of identified genes in Human Protein Atlas database
